# Supplementary material for: Multiple photofluorochromic luminogens via catalyst-free alkene oxidative cleavage photoreaction for dynamic 4D codes encryption
Source: Nat Commun. 2024 May 31;15:4647. doi: 10.1038/s41467-024-49033-2 (PMC11143217; doi:10.1038/s41467-024-49033-2)
Supplement: Supplementary file 1 — Supplementary Information [file 41467_2024_49033_MOESM1_ESM.pdf]

# Supplementary Information

Multiple photofluorochromic luminogens via catalyst-free alkene oxidative cleavage photoreaction for dynamic 4D codes encryption

Lin Lu,[1,2,3] Bo Wu,[3] Xinyuan He,[4] Fen Zhao,[3] Xing Feng,[5] Dong Wang,[1] Zijie Qiu,[3] Ting Han,[1*] Zheng Zhao[3*] and Ben Zhong Tang[3,4*]

**Affiliations**

[1] Center for AIE Research, Shenzhen Key Laboratory of Polymer Science and Technology, Guangdong Research Center for Interfacial Engineering of Functional Materials, College of Materials Science and Engineering, Shenzhen University, Shenzhen 518060, China.

[2] College of Physics and Optoelectronic Engineering, Shenzhen University, Shenzhen 518060, China.

[3] School of Science and Engineering, Shenzhen Institute of Aggregate Science and Technology, The Chinese University of Hong Kong, Shenzhen (CUHK-Shenzhen), Guangdong 518172, China.

[4] Department of Chemistry, Hong Kong Branch of Chinese National Engineering Research Center for Tissue Restoration and Reconstruction, The Hong Kong University of Science and Technology, Clear Water Bay, Kowloon, Hong Kong 999077, China.

[5] School of Material and Energy, Guangdong University of Technology, Guangzhou 510006, China.

Corresponding author email address: tangbenz@cuhk.edu.cn; zhaozheng@cuhk.edu.cn; hanting@szu.edu.cn.

**Supplementary Methods**

**1. Materials and Instruments**

All chemicals and reagents were purchased from commercial sources and used as received without further purification. Polyethylene glycol (PEG, $M_w$= 100,000) and Polymethyl methacrylate (PMMA, $M_w$= 120,000) for photofluorochromic application were purchased from Sigma-Aldrich Corp. The solvents with spectrographic grade used for photophysical measurements were purchased from Energy Chemical.

$^1$H and $^{13}$C NMR spectra were measured on a Bruker Advance 500 MHz NMR spectrometer using tetramethylsilane (TMS, $\delta$ = 0) as an internal reference. High-resolution mass spectra (HRMS) were carried out on a GCT Premier CAB 048 mass spectrometer. Crystallographic data for the compound was collected on a Bruker APEX 2 CCD diffractometer. UV-vis absorption spectra were performed on a Lambda 365 spectrophotometer. Fluorescence spectra and CIE diagrams were recorded on Edinburgh FLS1000 Instruments. Absolute fluorescence quantum yields were measured using Hamamatsu absolute PL quantum yield spectrometer C11347 Quantaurus QY. The electrochemical measurements were performed on an M204 Multi Autolab electrochemical workstation. Cyclic voltammetry tests were measured using a glassy carbon as the working electrode, a saturated calomel electrode as the reference electrode, a platinum wire as the auxiliary electrode, and 0.1 M tetrabutylammonium perchlorate/DCM as the supporting electrolyte. The application of fluorescent images in solution aggregate state and film state and 4D codes were irradiated by a 365 nm lamp with 0.1 W/cm$^2$. All other related photoreaction tests including dynamic NMR spectra, UV-vis spectra, and emission spectra were irradiated by a 365 nm light with 0.25 W/cm$^2$ and carried out in daylight background. Fluorescence photographs and antibacterial test photographs were captured by iPhone13 camera.

**2. Theoretical Calculations**

Theoretical calculations were performed using the ORCA 5.0 quantum chemistry program package from the development team at the Max Planck Institute for Bioinorganic Chemistry. The ground state geometry optimizations were carried out at the B3LYP-D3/def2-SVP level and the excited state geometry optimizations were

carried out at the CAM-B3LYP-D3/def2-SVP level. Time-dependent density functional theory (TD-DFT) excited state calculations were carried out at the CAM-B3LYP-D3/def2-TZVP level.

## 3. Synthesis

### 3.1 Synthesis of TPE-2MO2NT.

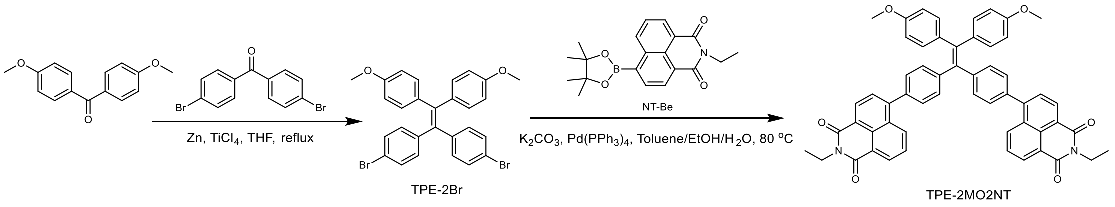


**Supplementary Figure 1. Synthetic route.** Synthetic route of TPE-2MO2NT.

4,4'-Dibromobenzophenone (1.70 g, 5 mmol), 4,4'-Dimethoxybenzophenone (1.21 g, 5 mmol), zinc powder (3.90 g, 60 mmol), and dry THF (60 mL) were added into 100 mL two-necked bottle under nitrogen atmosphere. $TiCl_4$ (3.30 mL, 30 mmol) was injected into the bottle at 0 °C, and the mixture was then refluxed for 24 h. The reaction was quenched by adding an ammonium chloride solution. The organic layer was extracted with ethyl acetate (EA) and brine three times. Then the collected organic phase was dried over anhydrous $Na_2SO_4$. After filtration and solvent evaporation, this crude product was purified by column chromatography using petroleum ether (PE)/DCM (10:1 v/v) as an eluent, and a white solid (TPE-2Br) was obtained in 40% yield (1.09 g). $^1$H NMR (500 MHz, $CDCl_3$) δ 7.24-7.19 (m, 4H), 6.93-6.83 (m, 8H), 6.68-6.62 (m, 4H), 3.76 (d, $J$ = 2.0 Hz, 6H). $^{13}$C NMR (126 MHz, $CDCl_3$) δ 158.28, 142.66, 141.36, 136.44, 135.47, 132.89, 132.38, 130.88, 120.16, 113.11, 55.01. HRMS: m/z calculated for $[M]^+$ $C_{28}H_{22}Br_2O_2$: 549.9968, found 549.9971.

TPE-2Br (0.55 g, 1 mmol), NT-Be (0.88 g, 2.5 mmol), $Pd(PPh_3)_4$ (57 mg, 0.05 mmol), and $K_2CO_3$ (0.83 g, 6 mmol) were added into 100 mL two-neck bottle under nitrogen atmosphere. And deoxygenated toluene/EtOH/$H_2O$ (28/8/4 mL) was injected into the bottle, and the mixture was stirred at 80 °C for 24 h. After the reaction, the solvent was removed under reduced pressure. The solid was dissolved in DCM and washed with brine and water. Then the organic layer was dried over anhydrous $Na_2SO_4$. After filtration and solvent evaporation, the crude product was purified by column chromatography using PE/DCM (1:5 v/v) as eluent, and a yellow solid (TPE-2MO2NT) was obtained in 70% yield (0.58 g). $^1$H NMR (500 MHz, $CDCl_3$) δ 8.67-8.62 (m, 4H),

8.24 (d, J = 8.5 Hz, 2H), 7.71 (t, J = 7.2 Hz, 4H), 7.31 (d, J = 12.9 Hz, 8H), 7.07 (d, J = 8.0 Hz, 4H), 6.74 (d, J = 8.2 Hz, 4H), 4.28 (q, J = 7.1 Hz, 4H), 3.81 (s, 6H), 1.39-1.32 (m, 6H). $^{13}$C NMR (126 MHz, $CDCl_3$) δ 163.96, 163.77, 158.41, 146.48, 144.26, 142.01, 137.54, 136.48, 135.77, 132.62, 132.42, 131.57, 130.94, 130.59, 129.81, 129.35, 128.55, 127.57, 126.57, 122.81, 121.52, 113.05, 55.06, 35.37, 13.23. HRMS: m/z calculated for $[M+H]^+$ $C_{56}H_{43}N_2O_6$: 839.3121, found 839.3104.

**3.2 Photosynthesis of BP-2NT and BP-2MO**.

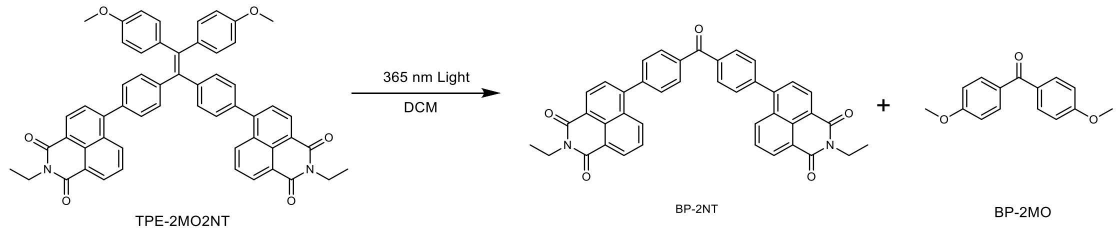


**Supplementary Figure 2. Synthetic route.** Photoreaction synthetic route of BP-2NT and BP-2MO.

TPE-2MO2NT (20 mg/20 mL) dissolved in DCM solution was added into a 100 mL beaker. The resulting solution was stirred under irradiation from a 100 W lamp for 10 h for a complete reaction. The crude product was purified by silica gel column chromatography with PE: DCM (1/1, v/v) to obtain two compounds BP-2NT (yield: 12.3 mg, 82.3%) and BP-2MO (yield: 4.51 mg, 78.1%). BP-2NT: $^1$H NMR (500 MHz, $CDCl_3$) δ 8.73-8.65 (m, 4H), 8.30-8.25 (m, 2H), 8.13-8.07 (m, 4H), 7.80-7.67 (m, 8H), 4.33-4.25 (m, 4H), 1.40-1.33 (m, 6H). $^{13}$C NMR (126 MHz, $CDCl_3$) δ 195.83, 164.27, 164.08, 145.67, 143.60, 137.60, 132.39, 131.66, 131.00, 130.76, 130.43, 130.12, 128.99, 128.22, 127.57, 123.45, 122.85, 35.95, 13.72. HRMS: m/z calculated for $[M+H]^+$ $C_{41}H_{29}N_2O_5$: 629.2076, found 629.2077. BP-2MO: $^1$H NMR (500 MHz, $CDCl_3$) δ 7.79 (d, *J* = 8.3 Hz, 4H), 6.96 (d, *J* = 8.3 Hz, 4H), 3.89 (s, 6H). $^{13}$C NMR (126 MHz, $CDCl_3$) δ 194.82, 163.22, 132.60, 131.16, 113.84, 55.85. HRMS: m/z calculated for $[M+H]^+$ $C_{15}H_{15}O_3$: 243.1021, found, 243.1019.

**3.3 Synthesis of TPE-4MO.**

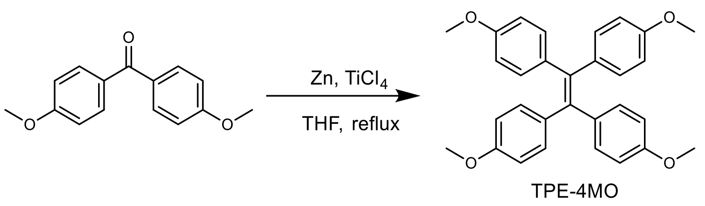


**Supplementary Figure 3. Synthetic route.** Synthetic route of TPE-4MO.

4,4'-Dimethoxybenzophenone (2.42 g, 10 mmol), zinc powder (3.90 g, 60 mmol), and dry THF (60 mL) were added into a 100 mL two-necked bottle under nitrogen atmosphere. $TiCl_4$ (3.30 mL, 30 mmol) was injected into the bottle at 0 °C, and the mixture was then refluxed for 24 h. The reaction was quenched by adding ammonium chloride solution. The organic layer was extracted with ethyl acetate (EA) and brine three times. Then the collected organic phase was dried over anhydrous $Na_2SO_4$. After filtration and solvent evaporation, this crude product was purified by column chromatography using PE/DCM (10:1 v/v) as an eluent and a white solid (TPE-4MO) was obtained in 80% yield (1.81 g). $^1$H NMR (500 MHz, $CDCl_3$) δ 6.92 (s, 8H), 6.64 (d, $J$ = 8.2 Hz, 8H), 3.75 (s, 12H). $^{13}$C NMR (126 MHz, $CDCl_3$) δ 158.17, 137.27, 132.95, 113.40, 55.46. HRMS: m/z calculated for $[M]^+$ $C_{30}H_{28}O_4$: 452.1988, found, 452.1990.

**3.4 Synthesis of TPE-4NT.**

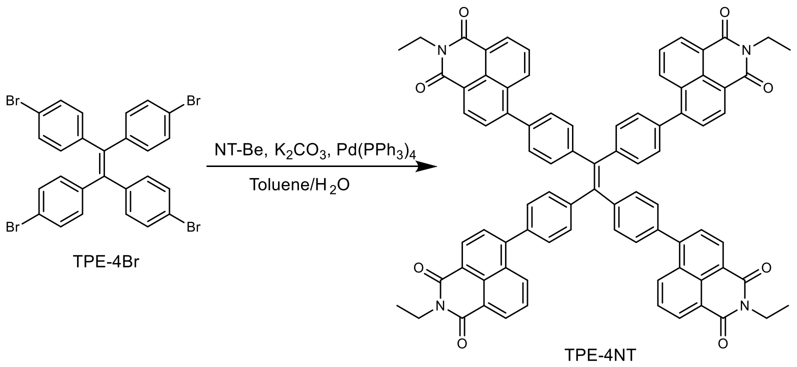


**Supplementary Figure 4. Synthetic route.** Synthetic route of TPE-4NT.

Compound TPE-4Br was synthesized by a similar method used to synthesize TPE-4MO. TPE-4Br (0.63 g, 1 mmol), NT-Be (1.76 g, 5 mmol), $Pd(PPh_3)_4$ (57 mg, 0.05 mmol), and $K_2CO_3$ (0.83 g, 6 mmol) were added into 100 mL two-neck bottle under nitrogen atmosphere. And deoxygenated toluene/EtOH/$H_2O$ (42/12/6 mL) was injected into the bottle, and the mixture was stirred at 80 °C for 24 h. After the reaction, the solvent was

removed under reduced pressure. The solid was dissolved in DCM and washed with brine and water. Then the organic layer was dried over anhydrous $Na_2SO_4$. After filtration and solvent evaporation, the crude product was purified by column chromatography using DCM as eluent, and a white solid (TPE-4NT) was obtained in 60% yield (0.73 g). $^1$H NMR (500 MHz, $CDCl_3$) δ 8.64 (dd, $J$ = 17.6, 7.4 Hz, 8H), 8.26 (d, $J$ = 8.6 Hz, 4H), 7.74 (d, $J$ = 7.6 Hz, 4H), 7.60 (t, $J$ = 7.9 Hz, 4H), 7.49-7.34 (m, 16H), 4.28 (q, $J$ = 7.3 Hz, 8H), 1.36 (t, $J$ = 7.1 Hz, 12H). $^{13}$C NMR (126 MHz, $CDCl_3$) δ 163.96, 163.77, 158.41, 146.48, 144.26, 142.01, 137.54, 136.48, 135.77, 132.62, 132.42, 131.57, 130.94, 130.59, 129.81, 129.35, 128.55, 127.57, 126.57, 122.81, 121.52, 113.05, 55.06, 35.37, 13.23. HRMS: m/z calculated for $[M+Na]^+$ $C_{82}H_{56}N_4O_8Na$: 1247.3995, found, 1247.3993.\

**3.5 Synthesis of TPE-2M2NT**

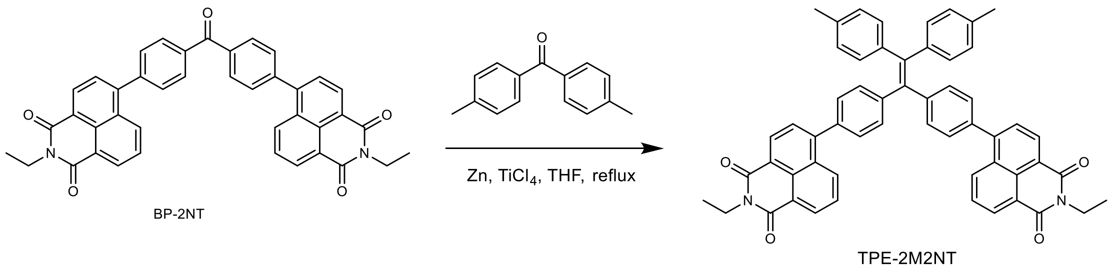


**Supplementary Figure 5. Synthetic route.** Synthetic route of TPE-2M2NT.

The synthesis of TPE-2M2NT in 18% yield (0.14 g) was similar to TPE-2Br. $^1$H NMR (500 MHz, $CDCl_3$) δ 8.63 (d, $J$ = 7.4 Hz, 4H), 8.23 (d, $J$ = 8.5 Hz, 2H), 7.70 (d, $J$ = 7.6 Hz, 4H), 7.31 (s, 8H), 7.02 (q, $J$ = 7.9 Hz, 8H), 4.27 (q, $J$ = 7.0 Hz, 4H), 2.39 (s, 6H), 1.27 (d, $J$ = 15.3 Hz, 6H). $^{13}$C NMR (126 MHz, $CDCl_3$) δ 164.26, 164.07, 146.78, 146.73, 144.32, 143.00, 140.68, 138.61, 136.89, 136.78, 132.72, 131.79, 131.49, 131.22, 130.88, 130.13, 129.57, 128.85, 128.67, 127.85, 126.83, 123.11, 121.83, 35.66, 21.43, 13.50. HRMS: m/z calculated for $[M+H]^+$ $C_{57}H_{42}N_2O_4$: 807.3223, found, 807.3221.

**3.6 Synthesis of TPE-2MO2CN**

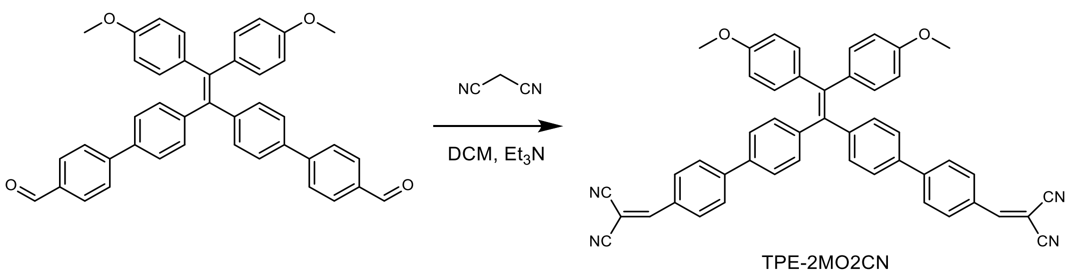


**Supplementary Figure 6. Synthetic route.** Synthetic route of TPE-2MO2CN.

Compound TPE-2MO2AB (0.6 g, 1 mmol) and malononitrile (0.17 g, 2.5 mmol) were dissolved in dry DCM (25 mL), and one drop of triethylamine was added to the solution. The mixture was stirred at room temperature for 2 h. Then, the mixture was washed with water three times, and the organic layer was dried over anhydrous $Na_2SO_4$. After filtration and solvent evaporation, the crude product was purified by column chromatography using PE/DCM (1:2 v/v) as eluent and a red solid was obtained in 82% yield (0.57 g). $^1$H NMR (500 MHz, $CDCl_3$) δ 7.99-7.92 (m, 4H), 7.79-7.70 (m, 6H), 7.45 (d, $J$ = 8.3 Hz, 4H), 7.18 (d, $J$ = 8.1 Hz, 4H), 7.03-6.96 (m, 4H), 6.71-6.65 (m, 4H), 3.76 (s, 6H). $^{13}$C NMR (126 MHz, $CDCl_3$) δ 159.25, 158.61, 146.85, 145.27, 142.28, 136.40, 135.93, 132.78, 132.40, 131.51, 129.76, 127.74, 126.69, 114.08, 113.34, 112.99, 81.76, 55.24. HRMS: m/z calculated for $[M]^+$ $C_{48}H_{32}N_4O_2$: 696.2525, found, 696.2546.

**3.7 Synthesis of TPE-2MO2Ph.**

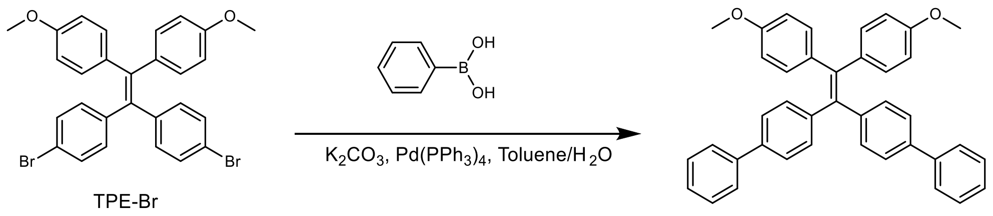


**Supplementary Figure 7. Synthetic route.** Synthetic route of TPE-2MO2Ph.

The synthesis of TPE-2MO2Ph in 70% yield (0.38 g) was similar to TPE-2MO2NT. $^1$H NMR (500 MHz, $CDCl_3$) δ 7.59 (d, $J$ = 7.7 Hz, 4H), 7.42 (t, $J$ = 8.3 Hz, 8H), 7.31 (t, $J$ = 7.3 Hz, 2H), 7.13 (s, 4H), 7.01 (d, $J$ = 8.2 Hz, 4H), 6.68 (d, $J$ = 8.5 Hz, 4H), 3.76 (s, 6H). $^{13}$C NMR (126 MHz, $CDCl_3$) δ 158.60, 141.04, 140.88, 136.80, 133.08, 132.38,

129.07, 127.51, 127.25, 126.58, 113.45, 55.48. HRMS: m/z calculated for $[M]^+$ $C_{40}H_{32}O_2$: 544.2402, found, 544.2402.

### 3.8 Synthesis of TPE-2MO2AB

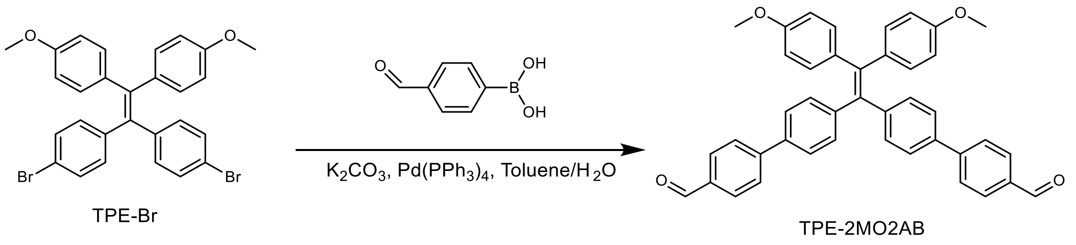


**Supplementary Figure 8. Synthetic route.** Synthetic route of TPE-2MO2AB.

The synthesis of TPE-2MO2AB in 72% yield (0.43 g) was similar to TPE-2MO2NT. $^1H$ NMR (500 MHz, $CDCl_3$) δ 10.03 (s, 2H), 7.95-7.88 (m, 4H), 7.76-7.69 (m, 4H), 7.47-7.41 (m, 4H), 7.17 (d, $J$ = 8.3 Hz, 4H), 7.04-6.97 (m, 4H), 6.70-6.65 (m, 4H), 3.75 (s, 6H). $^{13}C$ NMR (126 MHz, $CDCl_3$) δ 191.99, 158.51, 146.73, 144.71, 141.74, 137.73, 137.20, 136.11, 135.15, 132.78, 132.26, 130.35, 127.43, 126.77, 113.31, 55.23. HRMS: m/z calculated for $[M]^+$ $C_{42}H_{32}O_4$: 600.2300, found, 600.2301.

### 3.9 Synthesis of TPE-2MO2NB

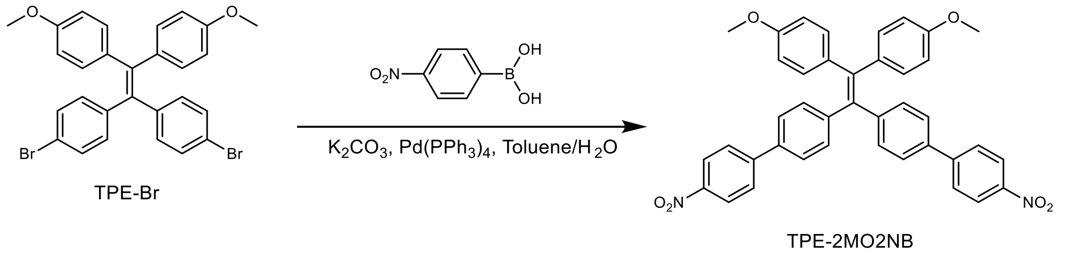


**Supplementary Figure 9. Synthetic route.** Synthetic route of TPE-2MO2NB.

The synthesis of TPE-2MO2NB in 70% yield (0.47 g) was similar to TPE-2MO2NT. $^1H$ NMR (500 MHz, $CDCl_3$) δ 8.31-8.21 (m, 4H), 7.75-7.67 (m, 4H), 7.43 (d, $J$ = 8.2 Hz, 4H), 7.18 (d, $J$ = 8.0 Hz, 4H), 7.04-6.95 (m, 4H), 6.73-6.63 (m, 4H), 3.76 (s, 6H). $^{13}C$ NMR (126 MHz, $CDCl_3$) δ 158.62, 147.15, 147.01, 145.14, 142.22, 136.31, 135.95, 132.79, 132.38, 127.53, 126.85, 124.19, 113.36, 55.25. HRMS: m/z calculated for $[M]^+$ $C_{40}H_{30}N_2O_6$: 634.2103, found, 634.2112.

### 3.10 Synthesis of TPE-MN-C5.

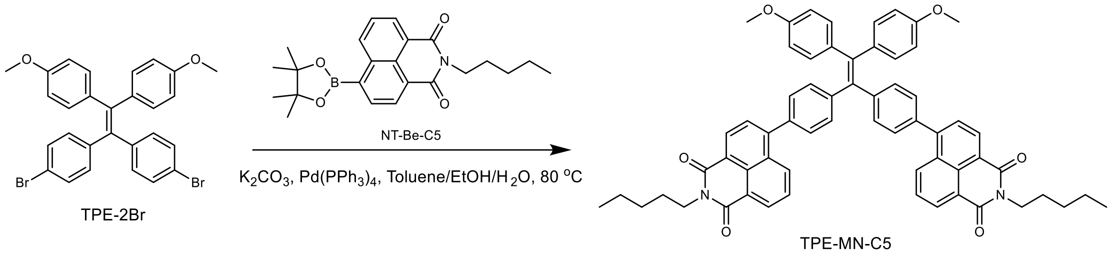


**Supplementary Figure 10. Synthetic route.** Synthetic route of TPE-MN-C5.

TPE-2Br (0.55 g, 1 mmol), NT-Be-C5 (0.98 g, 2.5 mmol), $Pd(PPh_3)_4$ (57 mg, 0.05 mmol), and $K_2CO_3$ (0.83 g, 6 mmol) were added into 100 mL two-neck bottle under nitrogen atmosphere. And deoxygenated toluene/EtOH/$H_2O$ (28/8/4 mL) was injected into the bottle, and the mixture was stirred at 80 °C for 24 h. After the reaction, the solvent was removed under reduced pressure. The solid was dissolved in DCM and washed with brine and water. Then the organic layer was dried over anhydrous $Na_2SO_4$. After filtration and solvent evaporation, the crude product was purified by column chromatography using PE/DCM (1:5 v/v) as eluent, and a yellow solid (TPE-MN-C5) was obtained in 72% yield (0.66 g). $^1$H NMR (500 MHz, $CDCl_3$) δ 8.63 (d, $J$ = 7.3 Hz, 4H), 8.24 (d, $J$ = 8.4 Hz, 2H), 7.71 (dt, $J$ = 7.8, 4.1 Hz, 4H), 7.32 (t, $J$ = 6.1 Hz, 8H), 7.07 (d, $J$ = 8.3 Hz, 4H), 6.78-6.70 (m, 4H), 4.24-4.16 (m, 4H), 3.80 (s, 6H), 1.76 (p, $J$ = 7.4 Hz, 4H), 1.42 (tt, $J$ = 8.5, 4.7 Hz, 8H), 0.92 (t, $J$ = 6.9 Hz, 6H). $^{13}$C NMR (126 MHz, $CDCl_3$) δ 164.30, 164.11, 158.59, 146.62, 144.42, 142.19, 137.72, 136.68, 135.94, 132.78, 132.54, 131.73, 131.12, 130.78, 129.99, 129.50, 128.74, 127.74, 126.74, 122.99, 121.71, 113.22, 55.22, 53.45, 40.50, 29.29, 27.83, 22.48, 14.03. HRMS: m/z calculated for $[M+H]^+$ $C_{62}H_{54}N_2O_6$: 923.4060, found 923.4077.

### 3.11 Synthesis of TPE-MN-Br.

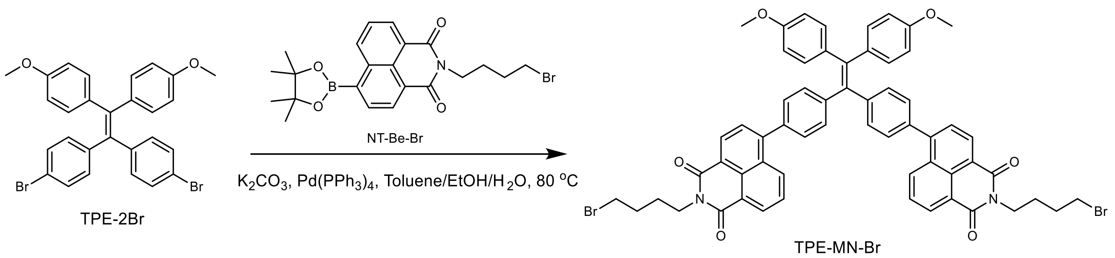

**Supplementary Figure 11. Synthetic route.** Synthetic route of TPE-MN-Br.

TPE-2Br (0.55 g, 1 mmol), NT-Be-Br (1.44 g, 2.5 mmol), $Pd(PPh_3)_4$ (57 mg, 0.05 mmol), and $K_2CO_3$ (0.83 g, 6 mmol) were added into 100 mL two-neck bottle under nitrogen atmosphere. And deoxygenated toluene/EtOH/$H_2O$ (28/8/4 mL) was injected into the bottle, and the mixture was stirred at 80 °C for 24 h. After the reaction, the solvent was removed under reduced pressure. The solid was dissolved in DCM and washed with brine and water. Then the organic layer was dried over anhydrous $Na_2SO_4$. After filtration and solvent evaporation, the crude product was purified by column chromatography using PE/DCM (1:5 v/v) as eluent, and a yellow solid (TPE-MN-Br) was obtained in 65% yield (0.69 g). $^1$H NMR (500 MHz, $CDCl_3$) δ 8.64 (dd, $J$ = 7.4, 1.6 Hz, 4H), 8.25 (dd, $J$ = 8.5, 1.2 Hz, 2H), 7.71 (dt, $J$ = 7.2, 4.1 Hz, 4H), 7.35-7.28 (m, 8H), 7.10-7.04 (m, 4H), 6.77-6.71 (m, 4H), 4.25 (t, $J$ = 7.1 Hz, 4H), 3.80 (s, 6H), 3.49 (t, $J$ = 6.6 Hz, 4H), 2.04-1.97 (m, 4H), 1.97-1.90 (m, 4H), 1.26 (s, 1H). $^{13}$C NMR (126 MHz, $CDCl_3$) δ 164.70, 164.51, 158.93, 147.19, 144.82, 142.58, 138.01, 136.95, 136.27, 133.14, 132.11, 131.65, 131.30, 130.36, 129.87, 129.11, 128.17 (d, $J$ = 5.4 Hz), 127.15, 123.14, 121.83, 113.57, 55.59, 39.76, 33.57, 30.64, 27.27. HRMS: m/z calculated for $[M]^+$ $C_{60}H_{48}Br_2N_2O_6$: 1052.1858, found 1052.1853.

**3.12 Synthesis of TPE-MN-TA.**

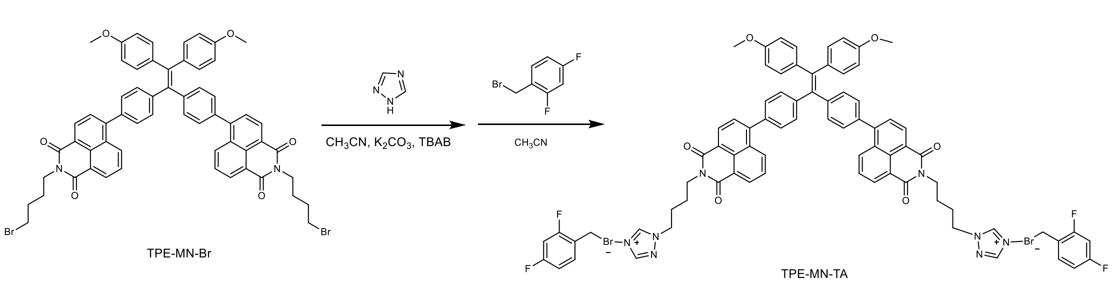


**Supplementary Figure 12. Synthetic route.** Synthetic route of TPE-MN-TA.

TPE-MN-Br (105 mg, 0.1 mmol), 1,2,4-triazole (20 mg, 0.25 mmol), $K_2CO_3$ (42 mg, 0.3 mmol), and TBAB (10 mg) were dissolved in $CH_3CN$/DCM (10/10mL). The mixture was stirred at 60 °C for 8 h under nitrogen atmosphere. After the reaction, the solvent was removed under reduced pressure. The solid was dissolved in DCM and washed with brine and water. Then the organic layer was dried over anhydrous $Na_2SO_4$.

After removing the solvent, the product was used directly without further purification. 1-(bromomethyl)-2,4-difluorobenzene (62 mg, 0.3 mmol) was added to a solution of the above obtained product (102 mg, 0.1 mmol) in $CH_3CN$ (20 mL). The mixture was stirred at 80 °C for 8 h under nitrogen atmosphere. After the reaction, the solvent was removed under reduced pressure. The crude product was purified by column chromatography using DCM as eluent, and an orange solid was obtained in 60% yield (86 mg). $^1$H NMR (500 MHz, DMSO) δ 10.23 (d, $J$ = 4.3 Hz, 2H), 9.33 (s, 2H), 8.52 (t, $J$ = 6.3 Hz, 4H), 8.17 (d, $J$ = 8.5 Hz, 2H), 7.86 (t, $J$ = 7.8 Hz, 2H), 7.79 (d, $J$ = 7.6 Hz, 2H), 7.69 (q, $J$ = 8.0 Hz, 2H), 7.40 (d, $J$ = 8.4 Hz, 6H), 7.29 (d, $J$ = 7.8 Hz, 4H), 7.24-7.18 (m, 2H), 7.01 (d, $J$ = 8.2 Hz, 4H), 6.81 (d, $J$ = 8.2 Hz, 4H), 5.57 (s, 4H), 4.43 (t, $J$ = 7.1 Hz, 4H), 4.10 (d, $J$ = 7.2 Hz, 4H), 3.73 (s, 6H), 1.94 (p, $J$ = 7.4 Hz, 4H), 1.69 (t, $J$ = 7.8 Hz, 4H). $^{13}$C NMR (126 MHz, DMSO) δ 164.16, 163.48, 162.08, 160.00, 158.31, 145.80, 144.90, 144.08, 143.07, 141.82, 137.63, 136.20, 135.50, 133.27, 132.45, 131.41, 129.46, 128.04, 122.44, 121.15, 117.31, 113.51, 112.38, 104.73, 55.28, 51.59, 44.53, 25.86, 24.43. HRMS: m/z calculated for $[M-2Br]^{2+}$ $C_{78}H_{62}F_4N_8O_6$: 641.2359, found: 641.2371.

**4. Antibacterial Test**

A single colony of bacteria (*S. aureus* and *E. coli*) on LB agar was transferred to 4 mL of LB liquid culture medium and grown at 37 °C with a shaking speed of 200 rpm overnight. Bacteria were harvested by centrifuging at 8000 rpm for 3 min and washed twice with PBS (pH = 7.4). After removal of the supernatant, the remaining bacteria were resuspended with PBS, and diluted to an optical density of 0.5 at 600 nm ($OD_{600}$ = 0.5 with about $10^8$ CFU mL $^{-1}$).

For the light-induced toxicity experiment, 1 mL ($10^6$ CFU mL$^{-1}$) bacteria (*S. aureus* and *E. coli*) were dispersed in the PBS solution containing AIEgens (0, 2, 5, 10 μM), and incubated at 37 °C with a shaking speed of 200 rpm for 10 min. Next, the bacterial suspensions were exposed to white light irradiation for 30 min (20 mW cm$^{-2}$) for phototoxicity test or were further incubated in the darkness at 37 °C to assess the dark toxicity. Afterward, the samples were diluted to a density of $10^3$ CFU mL$^{-1}$ with 1×PBS from which 100 μL ($10^3$ CFU mL$^{-1}$) bacteria were sprayed onto an LB agar plate,

followed by culturing at 37 °C for 16 h before CFU counting and taking photos.

**Supplementary Figures and Supplementary Tables**

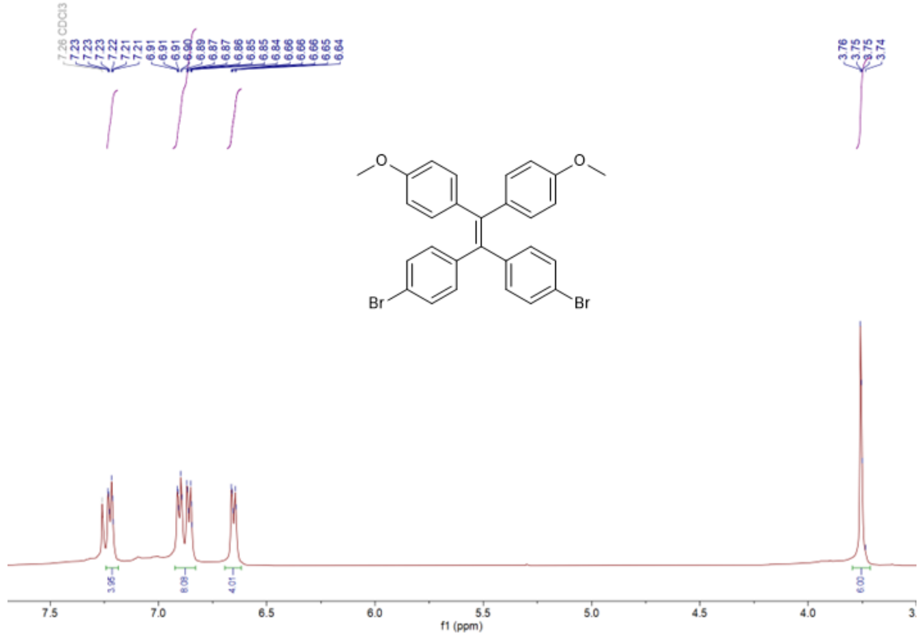


**Supplementary Figure 13. NMR spectrum.** $^{1}H$ NMR spectrum of TPE-2Br in $CDCl_3$.

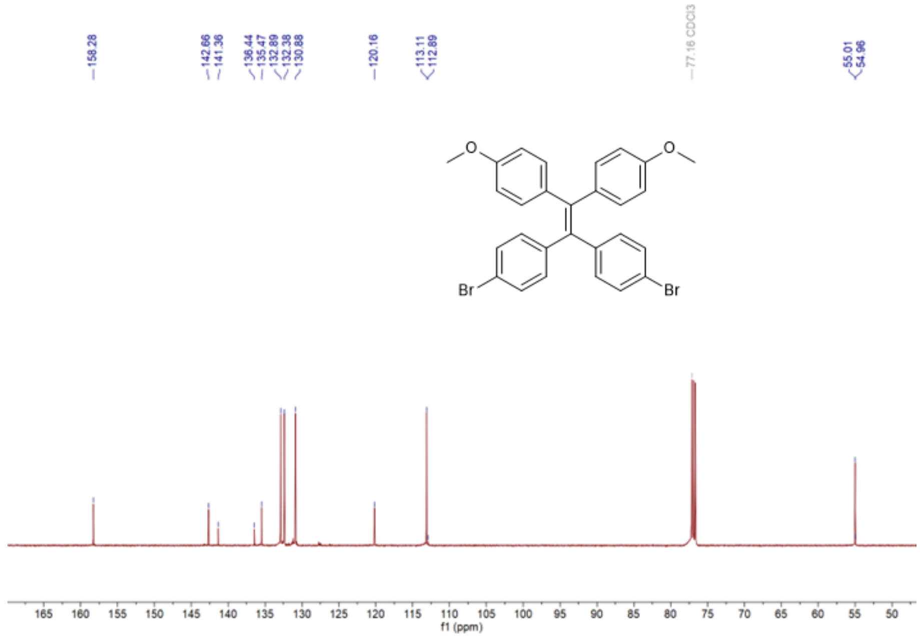


**Supplementary Figure 14. NMR spectrum.** $^{13}C$ NMR spectrum of TPE-2Br in $CDCl_3$.

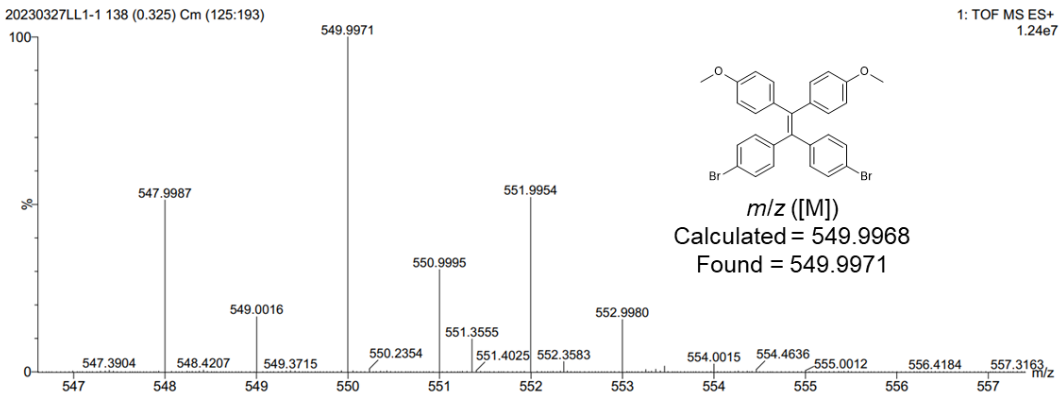


**Supplementary Figure 15. HRMS result.** High-resolution mass spectrum of TPE-2Br.

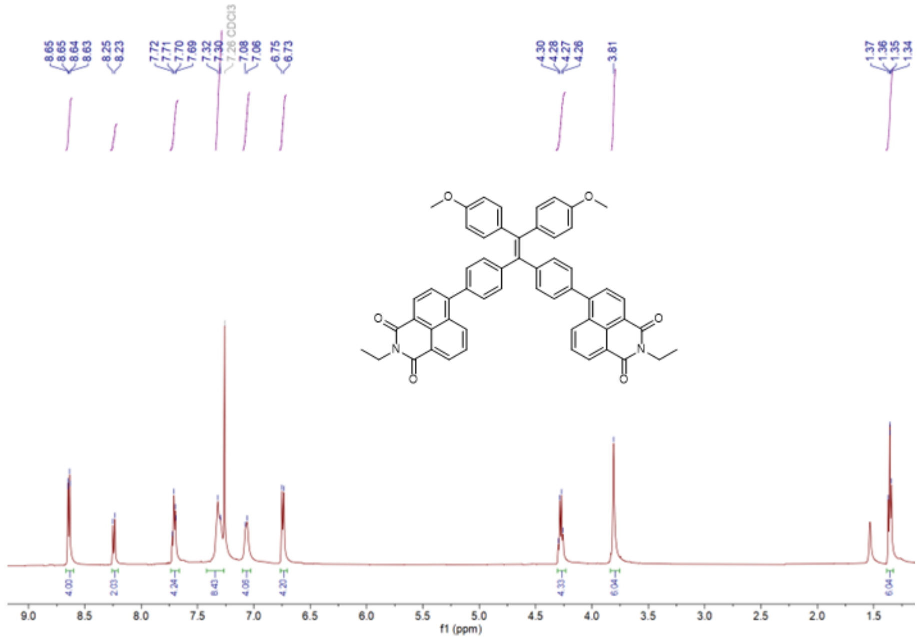


**Supplementary Figure 16. NMR spectrum.** $^{1}$H NMR spectrum of TPE-2MO2NT in $CDCl_3$.

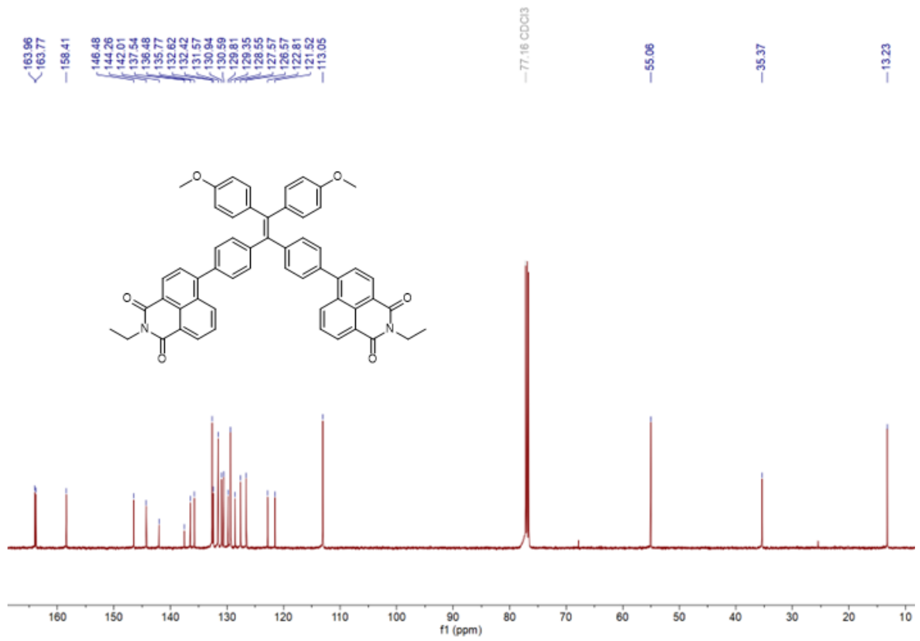


**Supplementary Figure 17. NMR spectrum.** $^{13}C$ NMR spectrum of TPE-2MO2NT in $CDCl_3$.

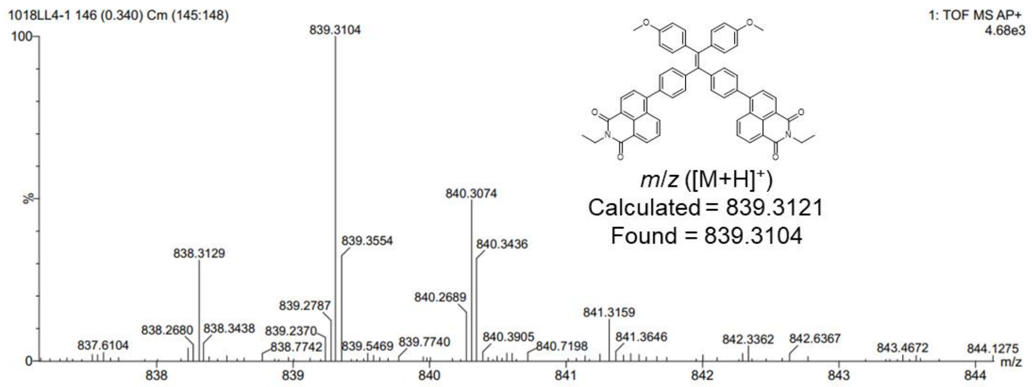


**Supplementary Figure 18. HRMS result.** High-resolution mass spectrum of TPE-2MO2NT.

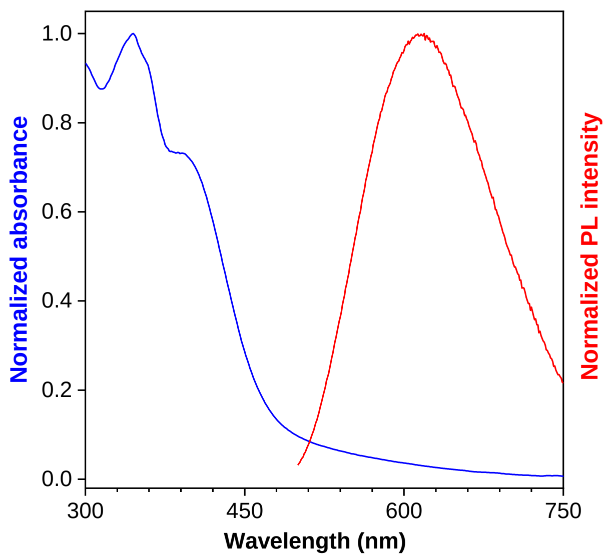


**Supplementary Figure 19. Photophysical test.** Absorption and PL spectra of TPE-2MO2NT in THF solution. Molecular concentration: $10^{-5}$ M; $\lambda_{ex}$ = 400 nm.

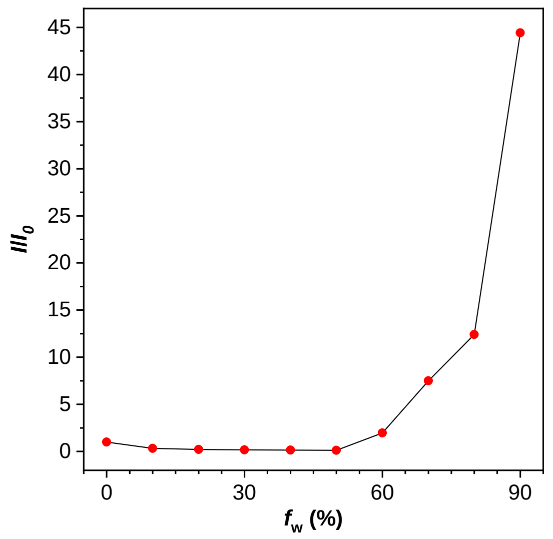


**Supplementary Figure 20. Relationship between PL intensity and water fractions.** Plots of relative PL intensity ($I/I_0$) of TPE-2MO2NT in THF/$H_2O$ solution-aggregates mixture versus water fractions. $I_0$ = emission intensity in THF ($f_w$ = 0%).

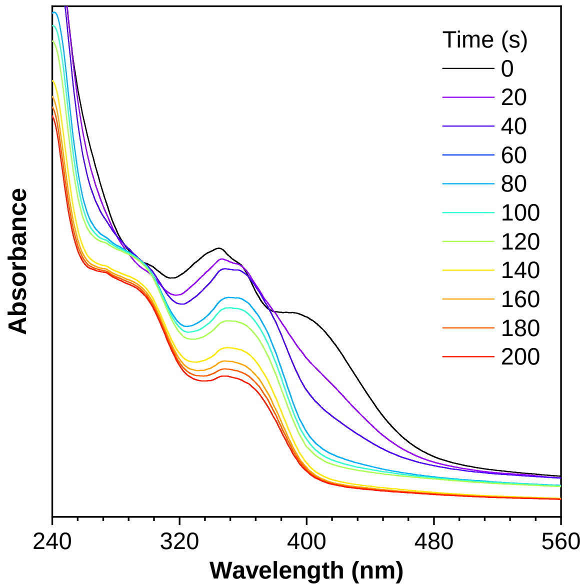


**Supplementary Figure 21. Absorption changes.** Absorption spectra of TPE-2MO2NT in THF/$H_2O$ ($f_w$ = 90%) aggregate mixtures over times upon 365 nm UV irradiation. Molecular concentration: $10^{-5}$ M.

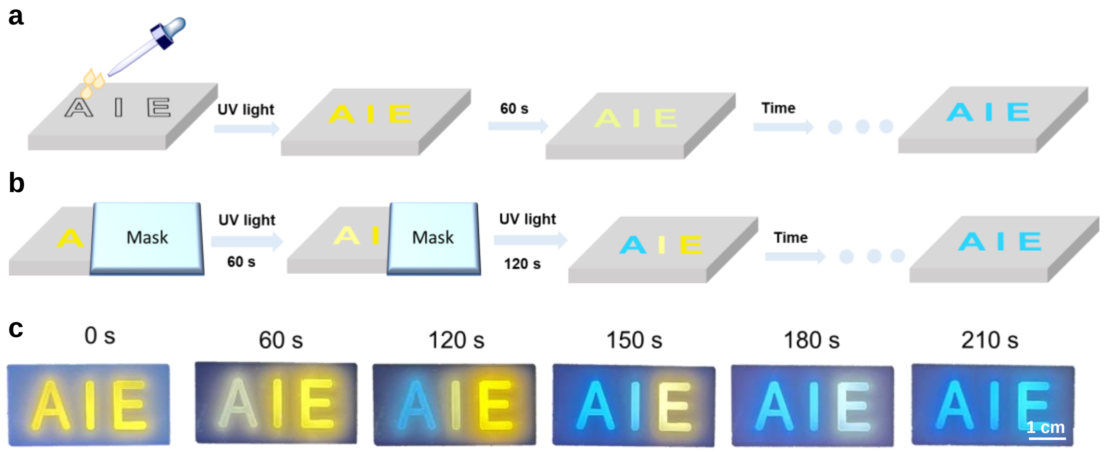


**Supplementary Figure 22. Application of the dynamic fluorescent pattern in solution state.** (a) Schematic diagram of the dynamic information AIE by adding TPE-2MO2NT in THF/$H_2O$ ($f_w$ = 90%) aggregate mixtures dropwise to Teflon containers. (b) Information fabrication process with a mask method. (c) Fluorescent images of multicolor information AIE made by UV light irradiated with a mask and all images share the same scale bar of 1 cm.

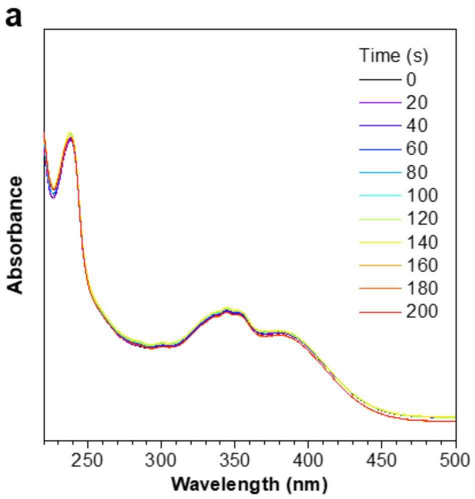


**Supplementary Figure 23. Photofluorochromic behavior in THF solution.** (a) UV-vis absorption and (b) PL spectra of TPE-2MO2NT in THF solution with different times upon 365 nm UV irradiation. Molecular concentration: $10^{-5}$ M; $\lambda_{ex}$ = 380 nm.

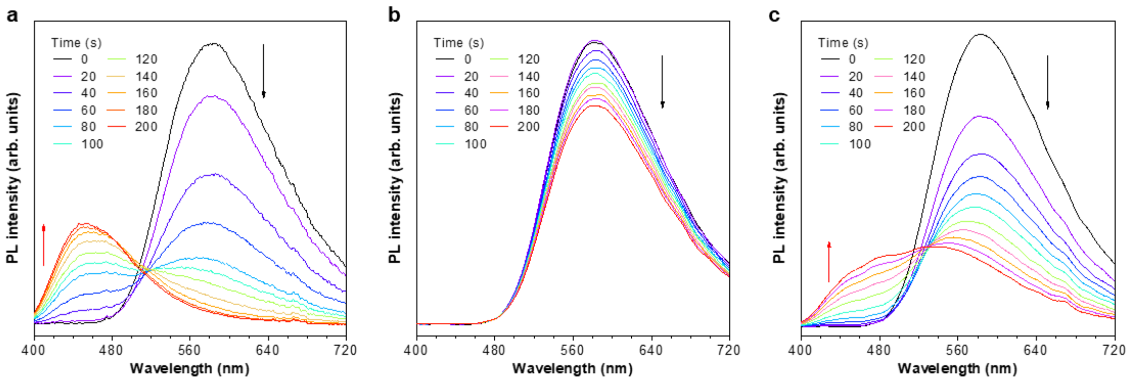


**Supplementary Figure 24. Photofluorochromic behavior with three different wavelengths of light irradiation.** PL spectra change of TPE-2MO2NT (10 µM) in THF/$H_2O$ ($f_w$ = 90%) aggregate mixtures with different times upon (a) 365 nm, (b) white light (400-700 nm) and (c) 405 nm light irradiation. $\lambda_{ex}$ = 380 nm.

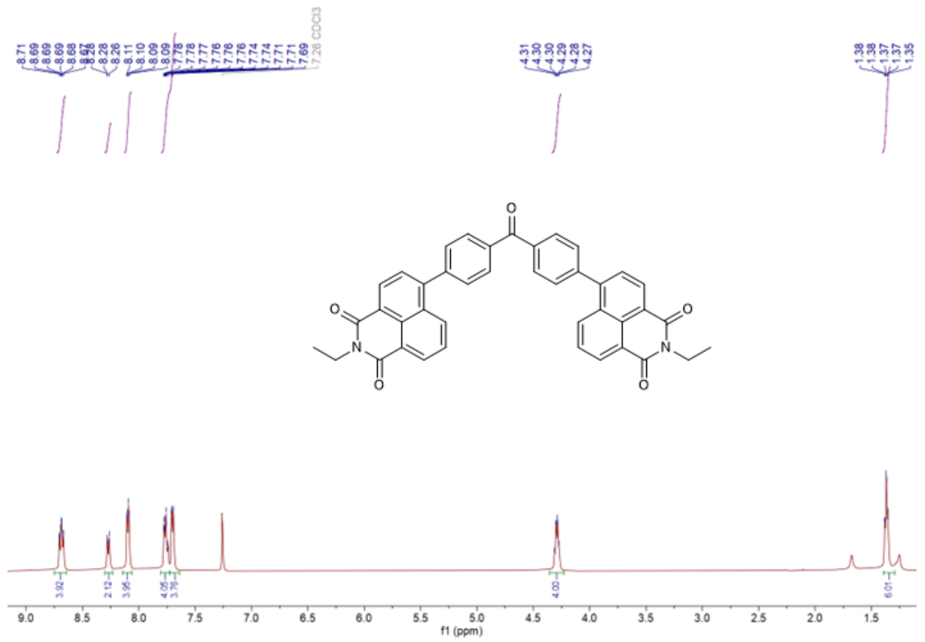

**Supplementary Figure 25. NMR spectrum.** $^{1}$H NMR spectrum of BP-2NT in $CDCl_3$.

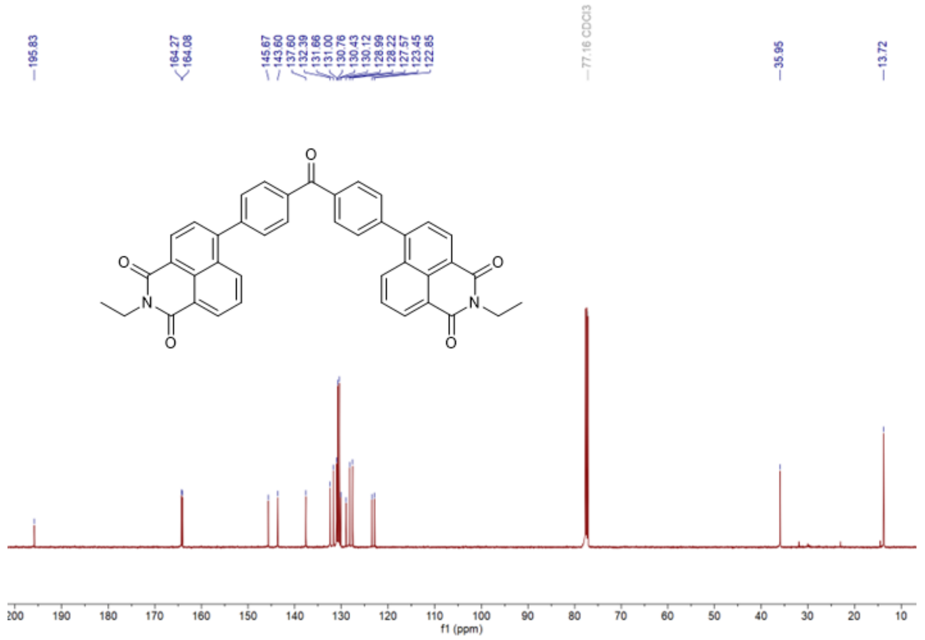

**Supplementary Figure 26. NMR spectrum.** $^{13}$C NMR spectrum of BP-2NT in $CDCl_3$.

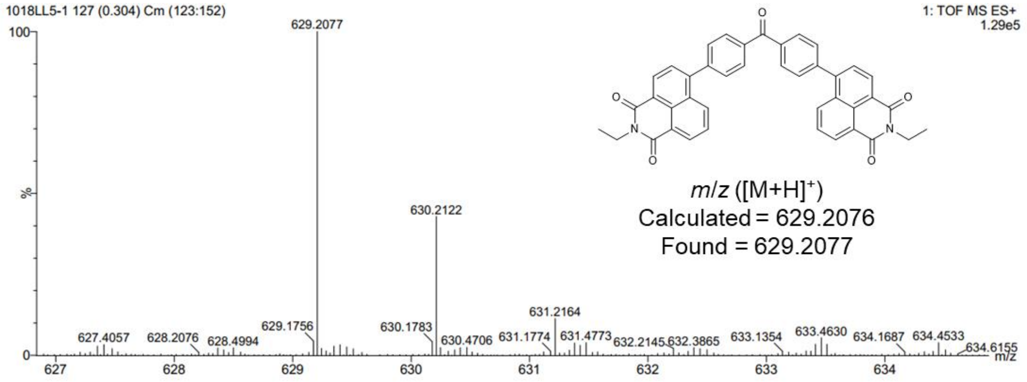


**Supplementary Figure 27. HRMS result.** High-resolution mass spectrum of BP-2NT.

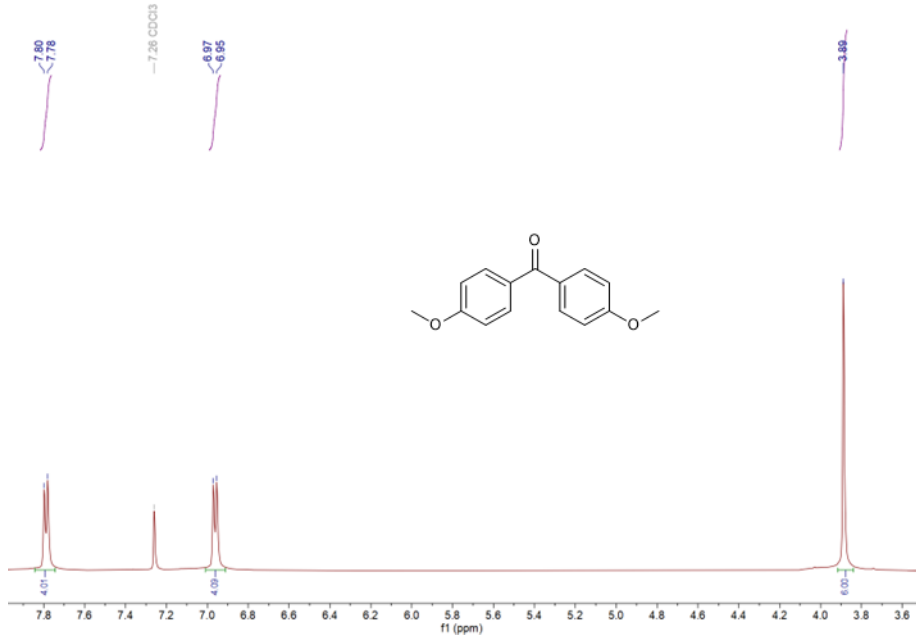


**Supplementary Figure 28. NMR spectrum.** $^{1}H$ NMR spectrum of BP-2MO in $CDCl_3$.

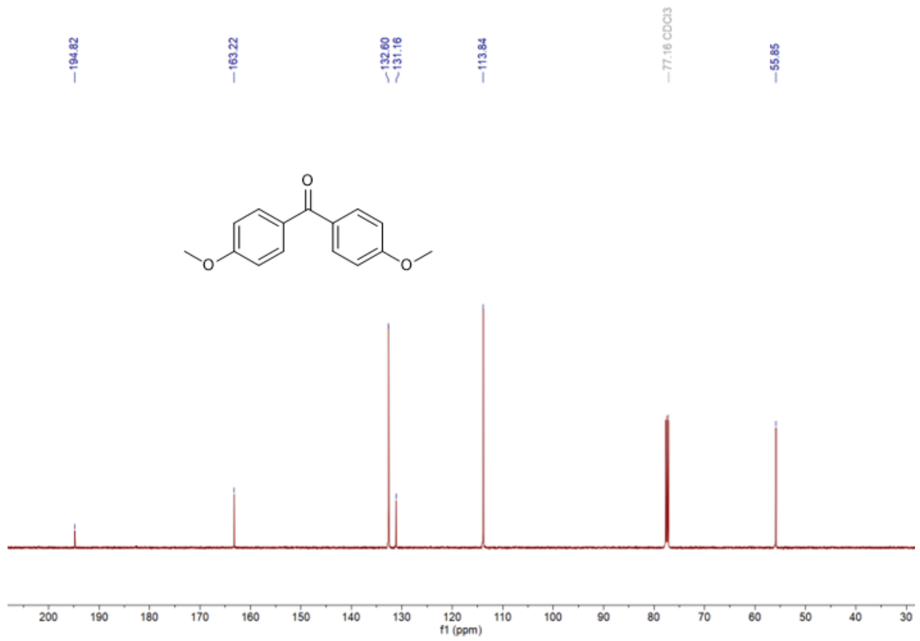


**Supplementary Figure 29. NMR spectrum.** $^{13}$C NMR spectrum of BP-2MO in $CDCl_3$.

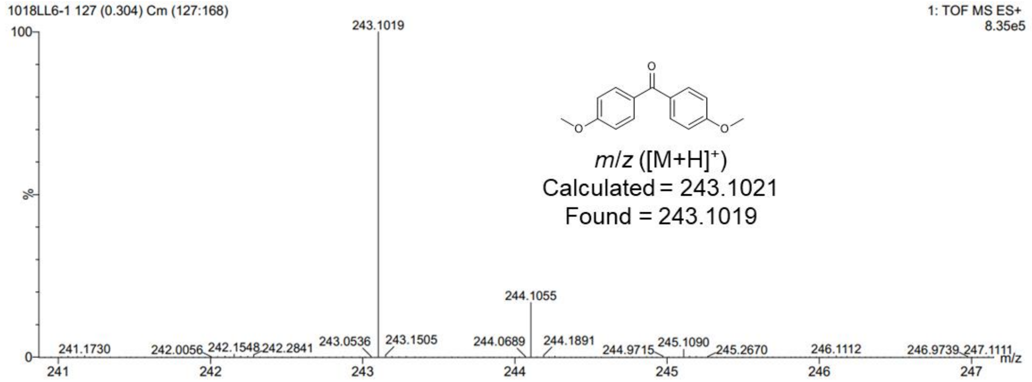


**Supplementary Figure 30. HRMS result.** High-resolution mass spectrum of BP-2MO.

**Supplementary Table 1. Crystal data and structure refinement for BP-2NT.**

| Complex | BP-2NT |
|---|---|
| Empirical formula | C41H28N2O5 |
| Formula weight | 628.65 |
| Crystal system | monoclinic |
| Space group | C2/c |
| a[Å] | 26.4491(7) |
| b[Å] | 18.6732(5) |
| c[Å] | 8.7579(2) |
| α[°] | 90 |
| β[°] | 99.1910(10) |
| γ[°] | 90 |
| Volume[$Å^3$] | 4269.90(19) |
| F(000) | 1312.0 |
| Z | 4 |
| Dcalcd[$Mg/m^3$] | 0.978 |
| temperature [K] | 239.98 |
| Radiation | MoKα (λ = 0.71073) |
| 2Θ range for data collection | 5.164 to 53.478 |
| Index ranges | -31 ≤ h ≤ 33, -23 ≤ k ≤ 16, -9 ≤ l ≤ 11 |
| Reflections collected | 7353 |
| Independent reflections | 3725 [Rint= 0.0430, Rsigma= 0.0588] |
| Data/restraints/parameters | 3725/0/219 |
| GOF on $F^2$ | 1.63 |
| CCDC | 2256098 |

**Supplementary Table 2. Crystal data and structure refinement for BP-2MO**.

| | |
|---|---|
| Complex | BP-2MO |
| Empirical formula | C15H14O3 |
| Formula weight | 242.26 |
| Crystal system | monoclinic |
| Space group | P-1 |
| a[Å] | 9.5574(6) |
| b[Å] | 9.6357(7) |
| c[Å] | 14.9218(11) |
| α[º] | 76.514(2)90 |
| β[º] | 78.167(2) |
| γ[º] | 70.141(2) |
| Volume[$Å^3$] | 1244.87(15) |
| F(000) | 512 |
| Z | 4 |
| Dcalcd[$Mg/m^3$] | 1.293 |
| temperature [K] | 300 |
| Radiation | MoKα (λ = 0.71073) |
| 2Θ range for data collection | 4.568 to 61.22 |
| Index ranges | -13 ≤ h ≤ 13, -12 ≤ k ≤ 13, -20 ≤ l ≤ 21 |
| Reflections collected | 17974 |
| Independent reflections | 7515 [Rint = 0.0568, Rsigma = 0.0727] |
| Data/restraints/parameters | 7515/0/329 |
| GOF on $F^2$ | 1.386 |
| CCDC | 2256097 |

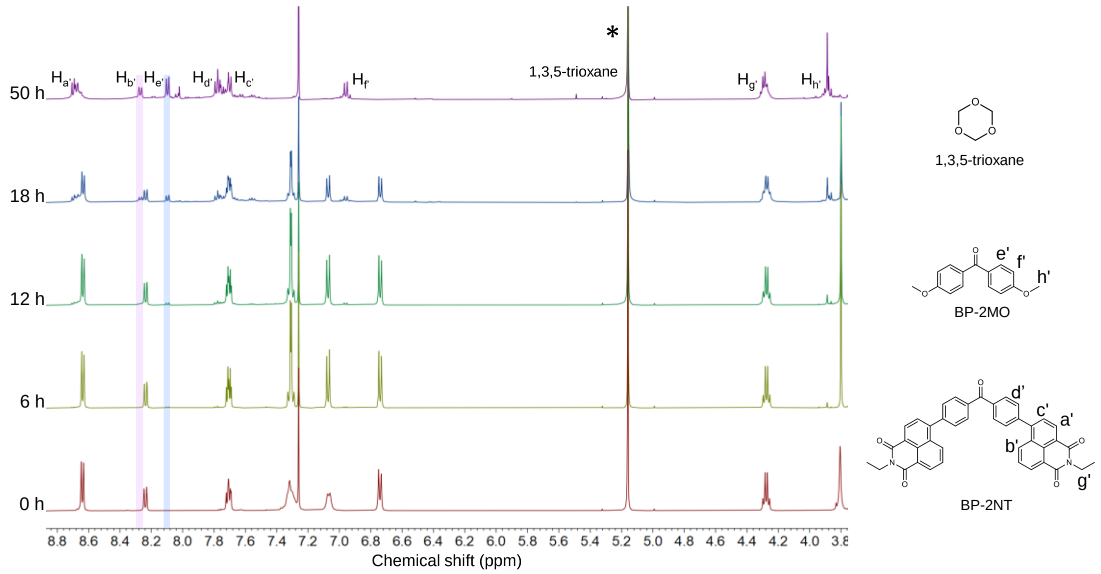


**Supplementary Figure 31. Photoconversions process.** $^1$H NMR spectra of TPE-2MO2NT (10 mM) in $CDCl_3$ solution under at different irradiation times and 1,3,5-trioxane was added as an internal standard. The $H_b$' proton signal of BP-2NT and $H_e$' proton signal of BP-2MO were the characteristic peaks, respectively. The integral area of the characteristic peak and 1,3,5-trioxane signal peak were used to calculate the reaction yield. $H_{e'}$, $H_{f'}$, and $H_{h'}$ are protons of BP-2MO. $H_{a'}$, $H_{b'}$, $H_{c'}$ $H_{d'}$ and $H_{g'}$ are protons of BP-2NT.

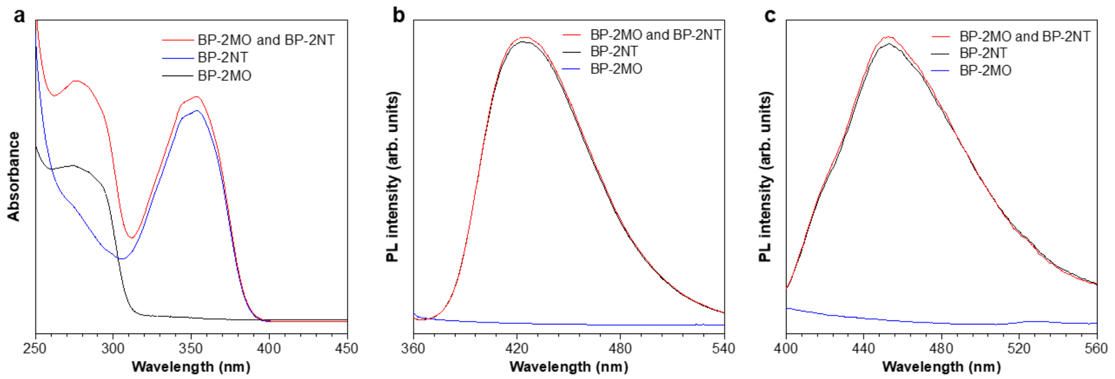


**Supplementary Figure 32. Photophysical properties of two photoproducts.** (a) Absorption spectra and (b) PL spectra of BP-2MO, BP-2NT, and the mixtures of BP-2MO and BP-2NT in the THF solution. (c) PL spectra of BP-2MO, BP-2NT, and the mixtures of BP-2MO and BP-2NT in THF/$H_2O$ aggregate mixtures with 90% water fractions. Molecular concentration: $10^{-5}$ M; $\lambda_{ex} = 340$ nm.

**Supplementary Table 3. Summary of photophysical data.** Photophysical properties of TPE-2MO2NT, BP-2NT, BP-2MO, and the mixtures of BP-2MO and BP-2NT in di fferent states.

| Compound | $\lambda_{abs}$ (nm) | $\lambda_{em}$ (nm) in THF soln | $\lambda_{em}$ (nm) in THF/$H_2O$ (90%) | Φ (%) in THF soln | Φ (%) in film |
|---|---|---|---|---|---|
| TPE-2MO2NT | 343; 377 | 615 | 582 | 2.2 | 32.9 |
| BP-2NT | 354 | 423 | 451 | 4.7 | 7.6 |
| BP-2MO | 285 | - | - | 0.01 | 0.03 |
| BP-2NT and BP-2MO | 285; 354 | 423 | 451 | 4.6 | 7.7 |

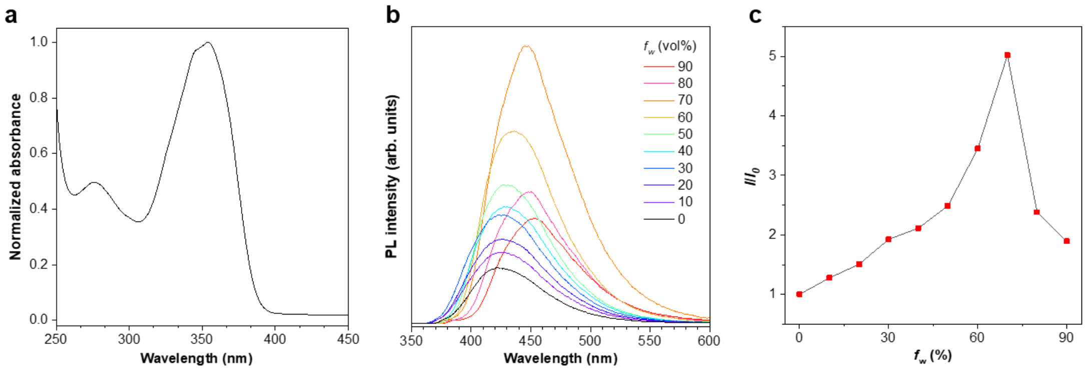


**Supplementary Figure 33. AIE behavior of BP-2NT.** (a) Absorption spectra of BP-2NT (10 μM) in THF solution. (b) PL spectra of BP-2NT in THF/$H_2O$ solution-aggregates mixtures with different water fractions. (c) Plots of relative PL intensity ($I/I_0$) versus water fractions. Molecular concentration: $10^{-5}$ M; $\lambda_{ex}$ = 340 nm; $I_0$ = emission intensity in THF ($f_w$ = 0%).

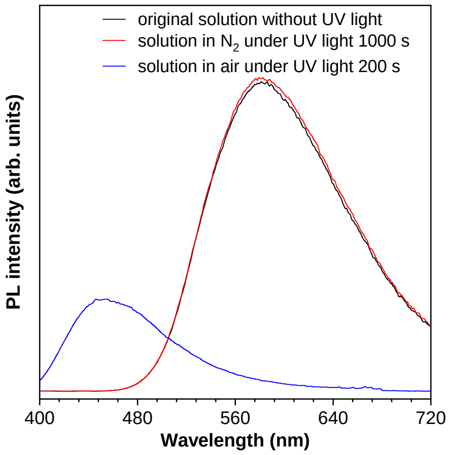

**Supplementary Figure 34. Control experiment in air or $N_2$ atmosphere.** PL spectra of TPE-2MO2NT in THF/$H_2O$ ($f_w$ = 90%) aggregate mixtures with three different conditions. Molecular concentration: $10^{-5}$ M. $\lambda_{ex}$ = 380 nm.

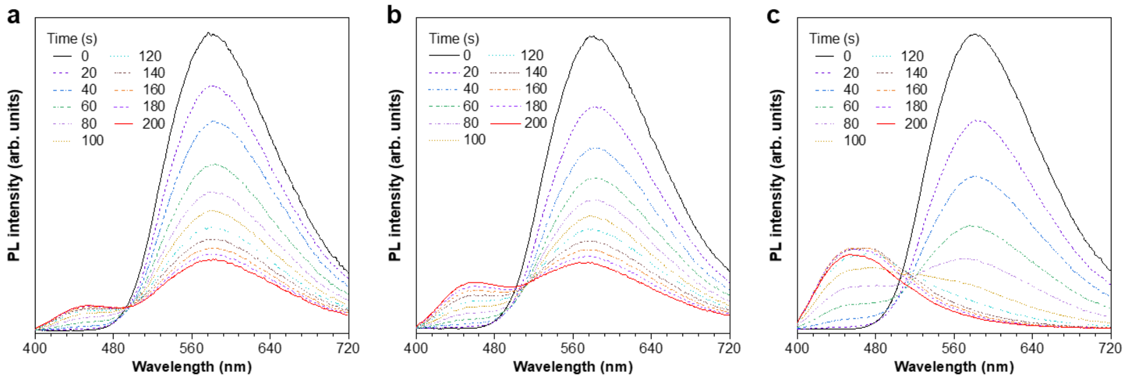


**Supplementary Figure 35. Control experiment with addition scavenger.** PL spectra of TPE-2MO2NT in THF/$H_2O$ ($f_w$ = 90%) aggregate mixtures with (a) benzoquinone (scavenger for $O_2^{\bullet-}$), (b) triethylenediamine (scavenger for $^1O_2$), and (c) tert-butyl alcohol (scavenger for •OH) after irradiation by a 365 nm UV light for different times. Molecular concentration: $10^{-5}$ M. $\lambda_{ex}$ = 380 nm.

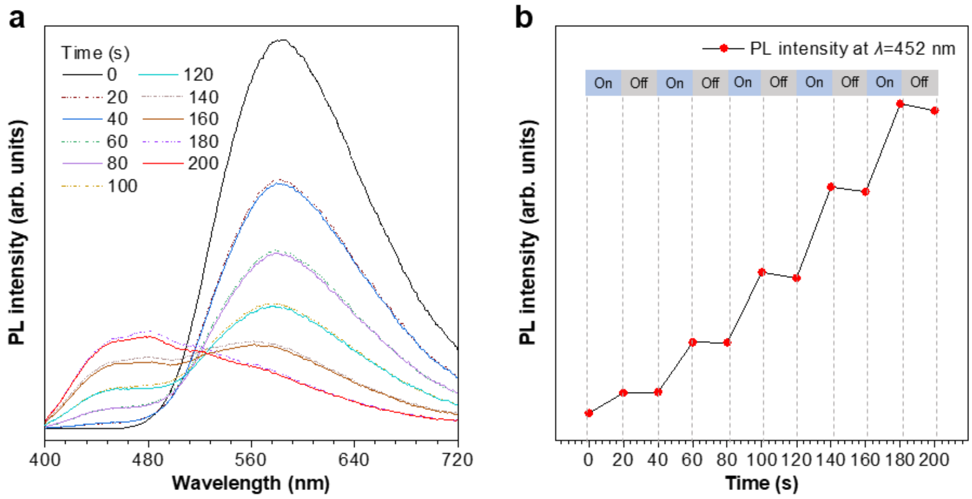


**Supplementary Figure 36. Control experiment with light on-off irradiation.** (a) PL spectra of TPE-2MO2NT in THF/$H_2O$ ($f_w$ = 90%) aggregate mixtures with different times upon 365 nm UV light on-off irradiation. (b) Change of PL intensity at $\lambda$ = 452 nm with light on-off experiments. Molecular concentration: $10^{-5}$ M. $\lambda_{ex}$ = 380 nm.

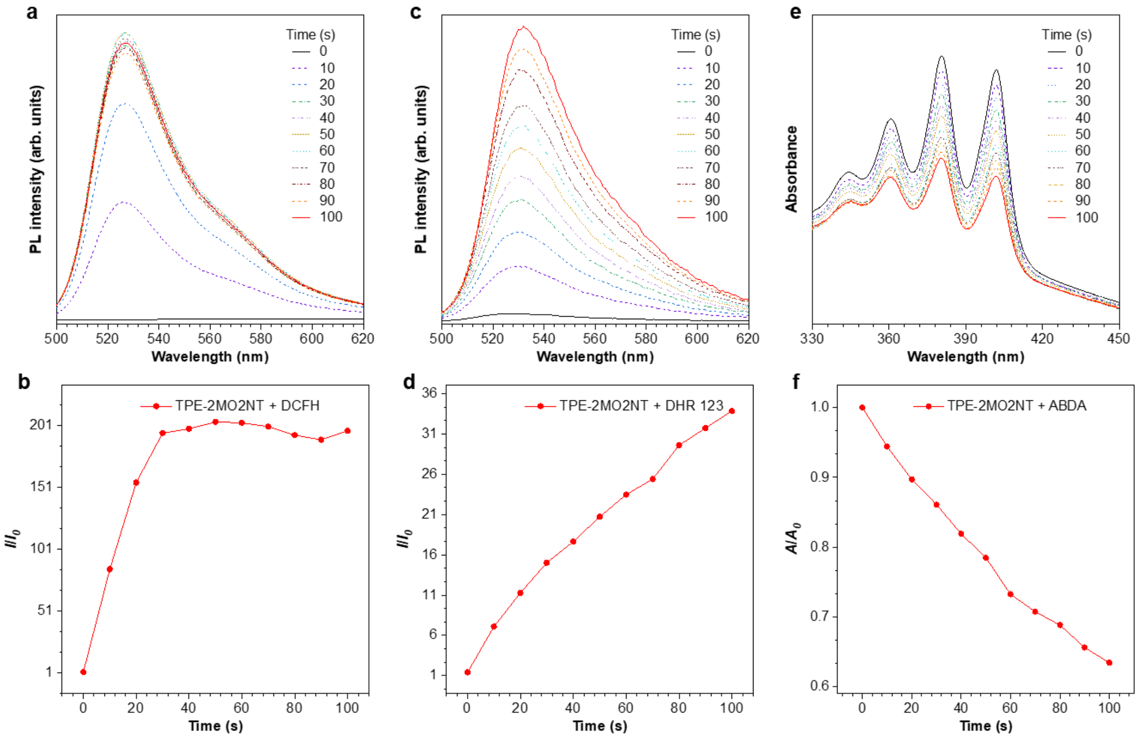


**Supplementary Figure 37. ROS generation ability of TPE-2MO2NT.** (a) PL spectra of DCFH (for overall ROS detection) in the presence of TPE-2MO2NT after exposure to UV light irradiation at different times. (b) Relative changes in PL intensity of DCFH with TPE-2MO2NT. (c) PL spectra of DHR 123 (for $O_2^{\bullet-}$ detection) in the presence of TPE-2MO2NT after exposure to UV light irradiation at different times. (d) Relative changes in PL intensity of DHR 123 with TPE-2MO2NT. (e) Absorption spectra of ABDA (for $^1O_2$ detection) in the presence of TPE-2MO2NT after exposure to UV light irradiation at different times. (f) Relative changes in absorbance of ABDA with TPE-2MO2NT.

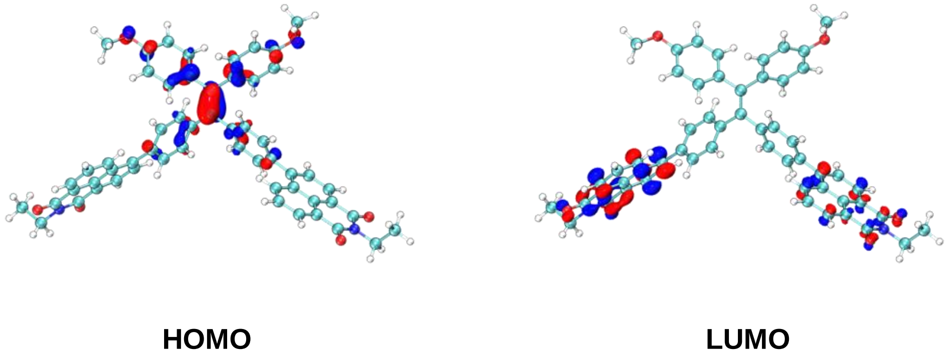

**Supplementary Figure 38. Optimized molecular orbital.** Molecular orbital amplitude plots of HOMO and LUMO of TPE-2MO2NT.

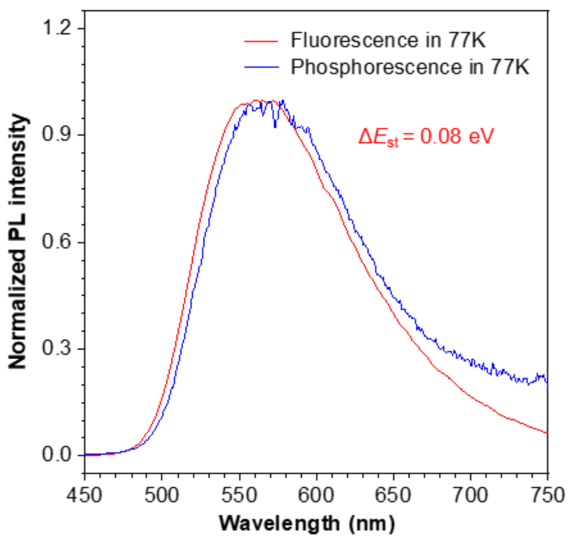


**Supplementary Figure 39. Luminescence spectra in 77K.** Fluorescence and phosphorescence spectra of TPE-2MO2NT powder in 77 K. $\lambda_{ex}$ = 380 nm.

**Supplementary Table 4. Summary data of theoretical calculation.** The singlet e nergy, triplet energy, singlet-triplet energy gap, and SOC coefficient values of TPE-2MO2NT.

| $T_n$ | Energy (eV) | $S_1^a/T_n$ | $S_1/T_n$ Energy gap | $\xi^b$ ($S_1$, $T_n$) ($cm^{-1}$) |
|---|---|---|---|---|
| T1 | 2.019 | $S_1/T_1$ | 1.264 | 0.168 |
| T2 | 2.069 | $S_1/T_2$ | 1.214 | 0.117 |
| T3 | 2.181 | $S_1/T_3$ | 1.102 | 0.227 |
| T4 | 3.039 | $S_1/T_4$ | 0.244 | 0.443 |
| T5 | 3.225 | $S_1/T_5$ | 0.058 | 0.453 |
| T6 | 3.25 | $S_1/T_6$ | 0.033 | 0.199 |
| T7 | 3.418 | $S_1/T_7$ | -0.135 | 0.127 |
| T8 | 3.424 | $S_1/T_8$ | -0.141 | 0.205 |
| T9 | 3.609 | $S_1/T_9$ | -0.326 | 0.115 |
| T10 | 3.649 | $S_1/T_{10}$ | -0.366 | 0.182 |

a: $S_1$ = 3.283 eV. b: SOC coefficients (ξ).

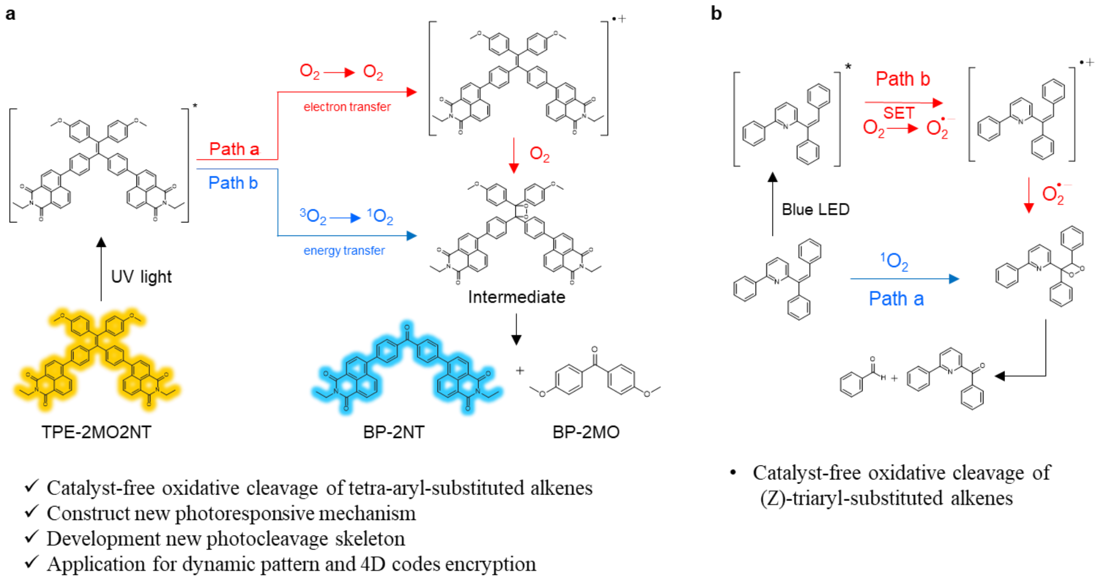


**Supplementary Figure 40. Different advantages of the two works.** Comparison of reaction mechanism and research focus of (a) our work and (b) previous reported work.

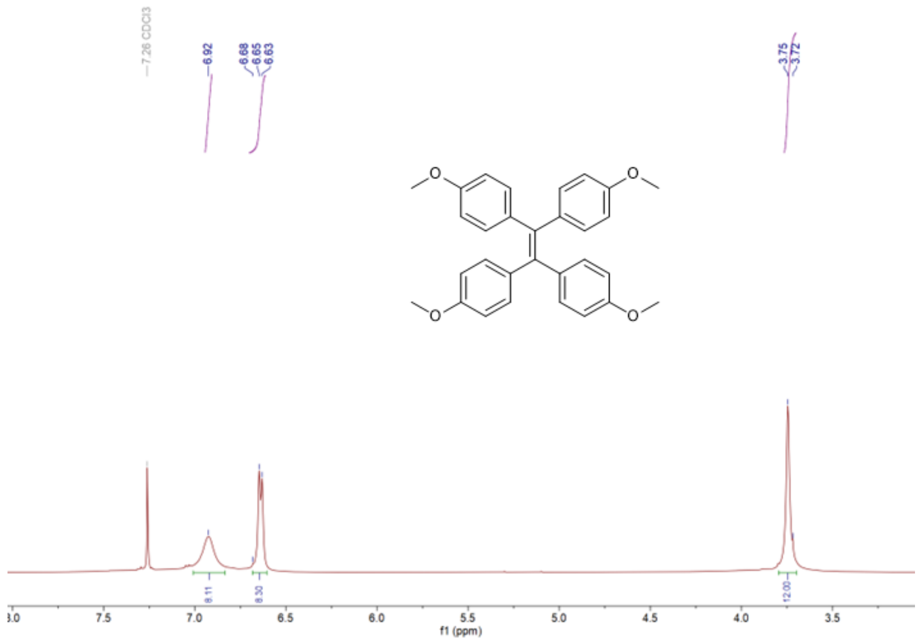


**Supplementary Figure 41. NMR spectrum.** $^{1}H$ NMR spectrum of TPE-4MO in $CDCl_3$.

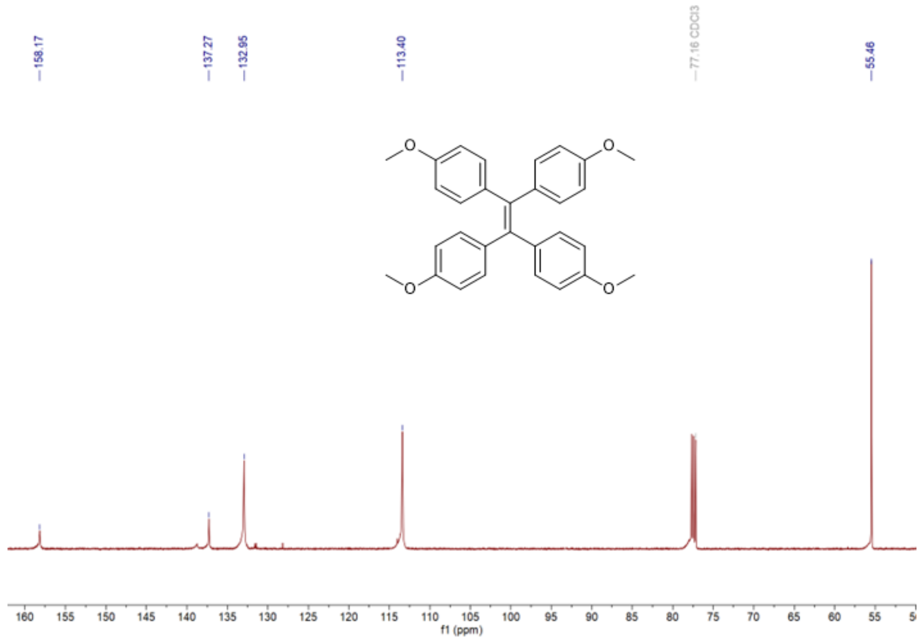


**Supplementary Figure 42. NMR spectrum.** $^{13}C$ NMR spectrum of TPE-4MO in $CDCl_3$.

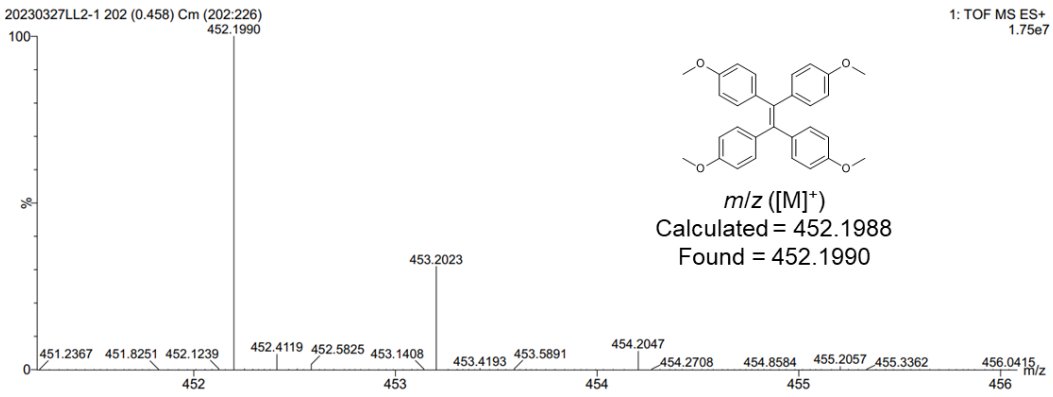


**Supplementary Figure 43. HRMS result.** High-resolution mass spectrum of TPE-4MO.

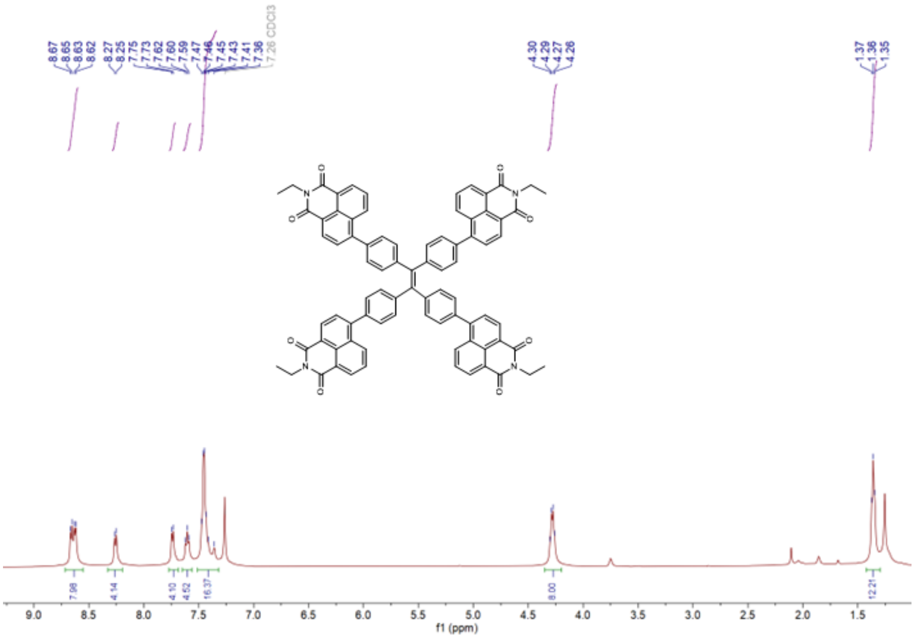


**Supplementary Figure 44. NMR spectrum.** $^{1}$H NMR spectrum of TPE-4NT in $CDCl_3$.

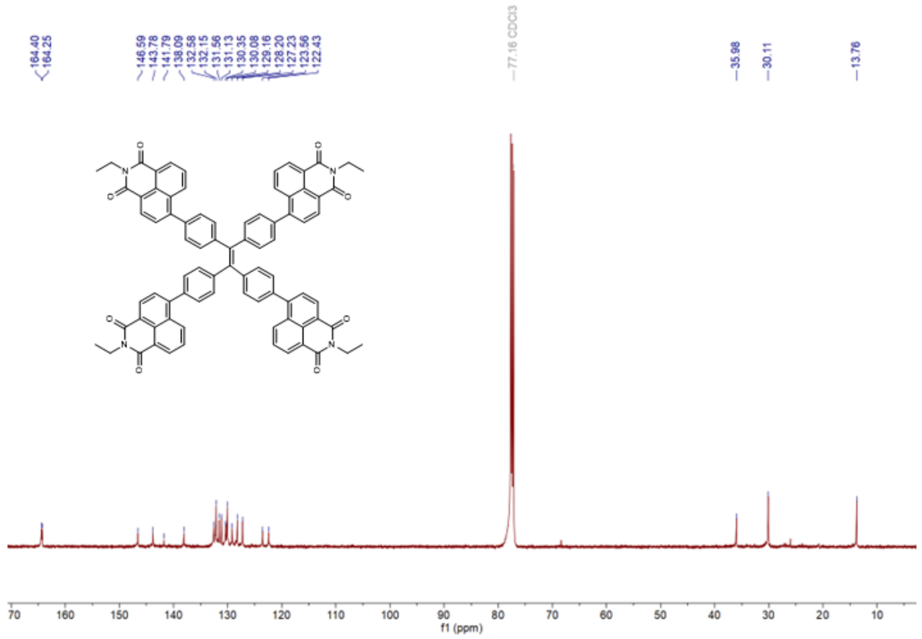


**Supplementary Figure 45. NMR spectrum.** $^{13}C$ NMR spectrum of TPE-4NT in $CDCl_3$.

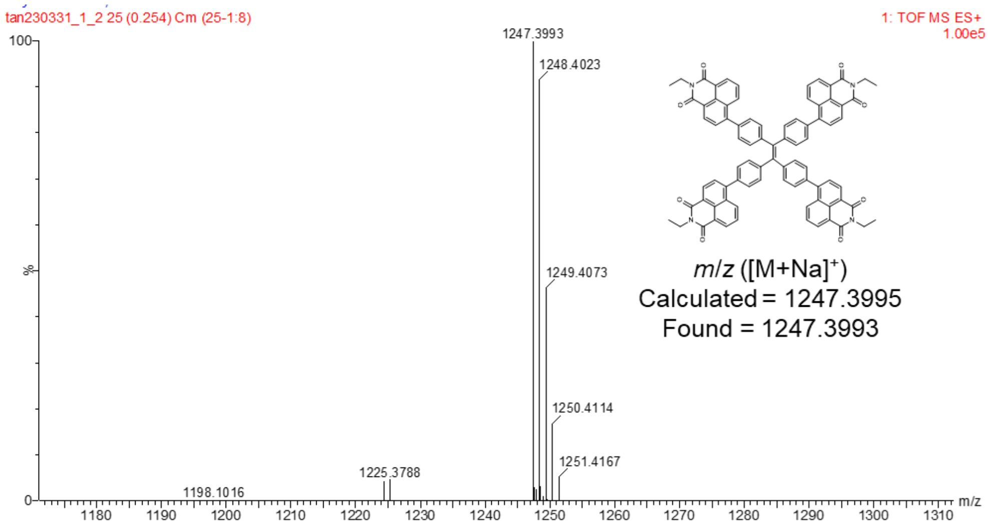


**Supplementary Figure 46. HRMS result.** High-resolution mass spectrum of TPE-4NT.

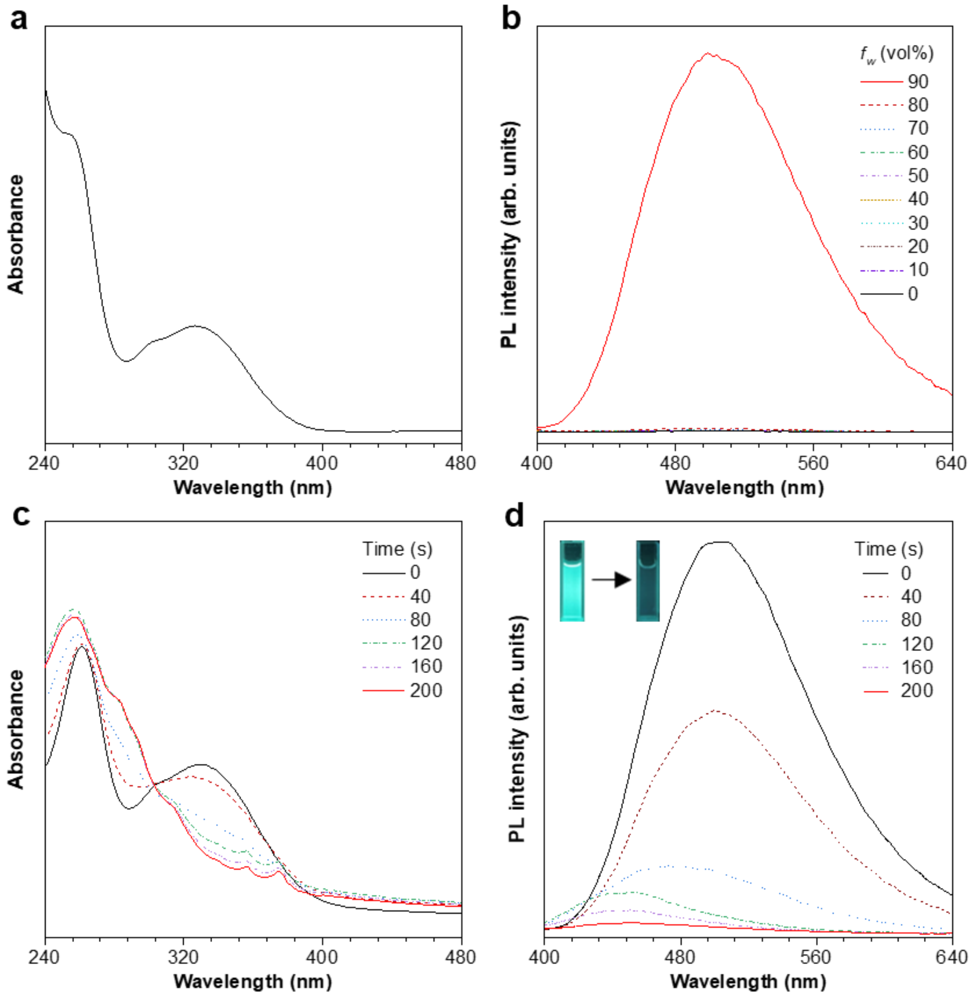


**Supplementary Figure 47. Photophysical property of TPE-4MO.** (a) The absorption spectrum of TPE-4MO in THF solution. (b) PL spectra of TPE-4MO in THF/$H_2O$ solution-aggregates mixture with different water fractions ($f_w$). (c) Absorption spectra of TPE-4MO in THF/$H_2O$ ($f_w$ = 90%) aggregate mixtures upon 365 nm UV irradiation. (d) PL spectra of TPE-4MO in THF/$H_2O$ ($f_w$ = 90%) aggregate mixtures with different times upon 365 nm UV irradiation. Inset: photographs of the TPE-4MO aggregates before and after UV irradiation for 200 s. Molecular concentration: $10^{-5}$ M; $\lambda_{ex}$ = 340 nm.

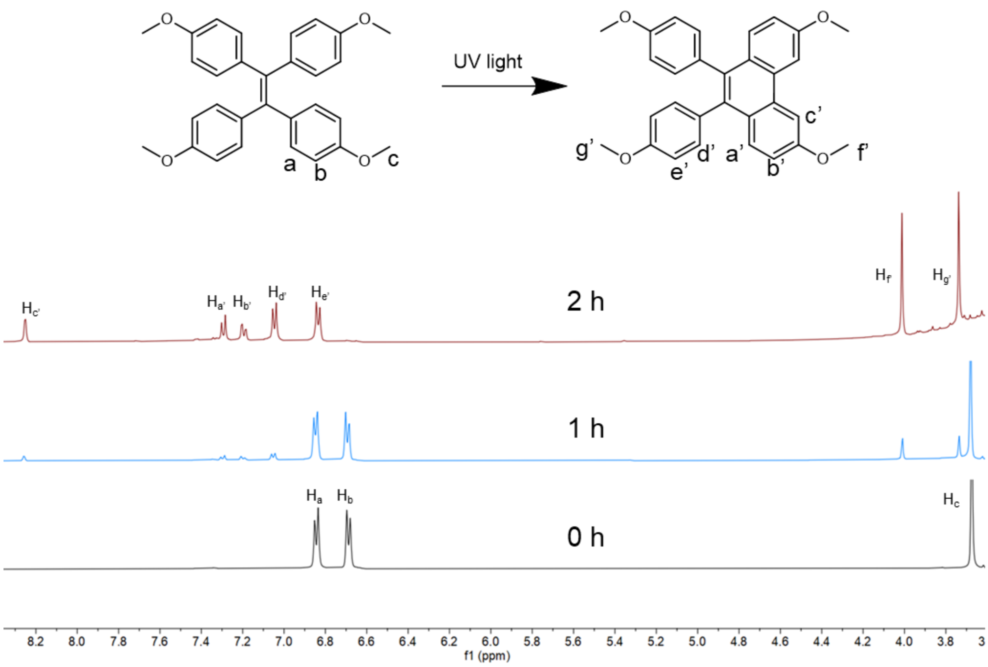


**Supplementary Figure 48. Dynamic NMR spectra in the illumination process.** Change of $^{1}H$ NMR spectra of TPE-4MO in DMSO-$d_6$ solution under different irradiation times.

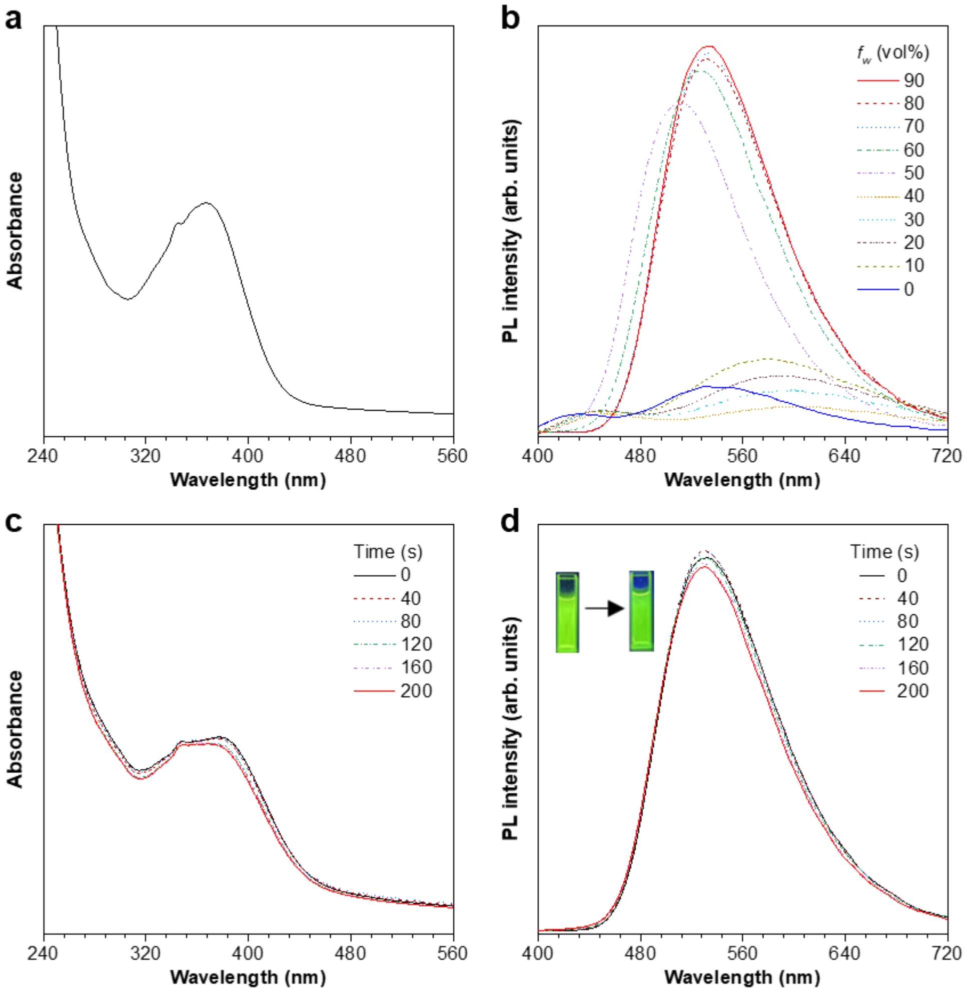


**Supplementary Figure 49. Photophysical property of TPE-4NT.** (a) The absorption spectrum of TPE-4NT in THF solution. (b) PL spectra of TPE-4NT in THF/$H_2O$ solution-aggregates mixture with different water fractions ($f_w$). (c) Absorption spectra of TPE-4NT in THF/$H_2O$ ($f_w$ = 90%) aggregate mixtures upon 365 nm UV irradiation. (d) PL spectra of TPE-4NT in THF/$H_2O$ ($f_w$ = 90%) aggregate mixtures with different times upon 365 nm UV irradiation. Inset: photographs of the TPE-4NT aggregates before and after UV irradiation for 200 s. Molecular concentration: $10^{-5}$ M; $\lambda_{ex}$ = 380 nm.

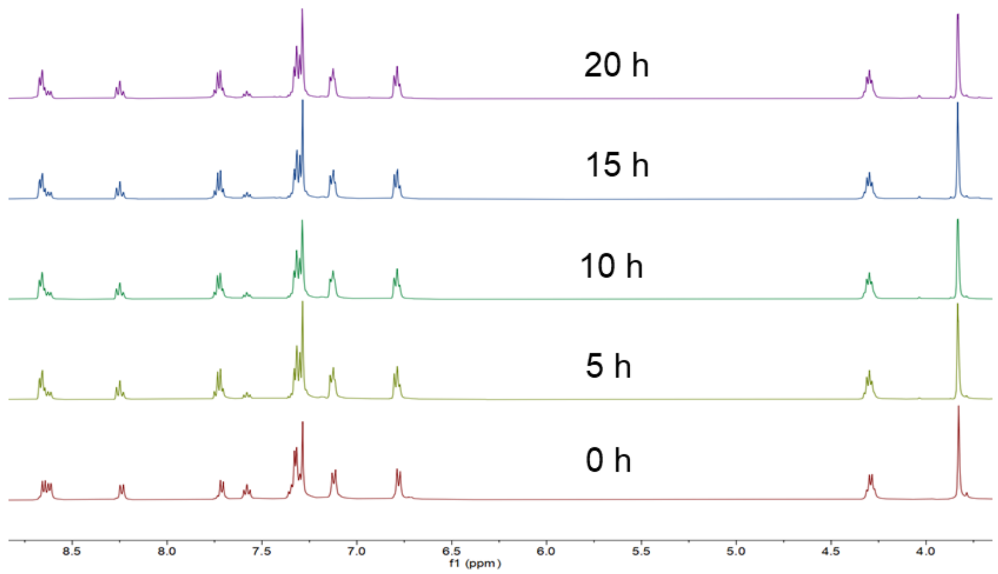


**Supplementary Figure 50. Dynamic NMR spectra in the illumination process.** $^{1}$H NMR spectra of TPE-4NT in $CDCl_3$ solution under different irradiation times.

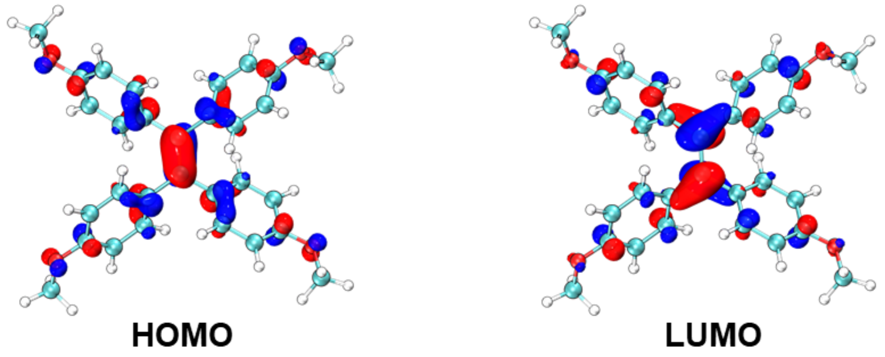


**Supplementary Figure 51. Optimized molecular orbital.** Molecular orbital amplitude plots of HOMO and LUMO of TPE-4MO.

**Supplementary Table 5. Summary data of theoretical calculation.** The singlet energy, triplet energy, singlet-triplet energy gap, and SOC coefficients values of TPE-4MO.

| $T_n$ | Energy (eV) | $S_1^a/T_n$ | $S_1/T_n$ Energy gap | $\xi^b$ ($S_1$, $T_n$ ) ($cm^{-1}$) |
|---|---|---|---|---|
| T1 | 2.164 | $S_1/T_1$ | 1.421 | 0.02 |
| T2 | 3.215 | $S_1/T_2$ | 0.37 | 0.657 |
| T3 | 3.227 | $S_1/T_3$ | 0.358 | 0.868 |
| T4 | 3.366 | $S_1/T_4$ | 0.219 | 0.024 |
| T5 | 3.709 | $S_1/T_5$ | -0.124 | 0.254 |
| T6 | 3.78 | $S_1/T_6$ | -0.195 | 0.216 |
| T7 | 3.902 | $S_1/T_7$ | -0.317 | 0.732 |
| T8 | 3.956 | $S_1/T_8$ | -0.371 | 0.145 |

a: $S_1$ = 3.585 eV; b: SOC coefficients (ξ).

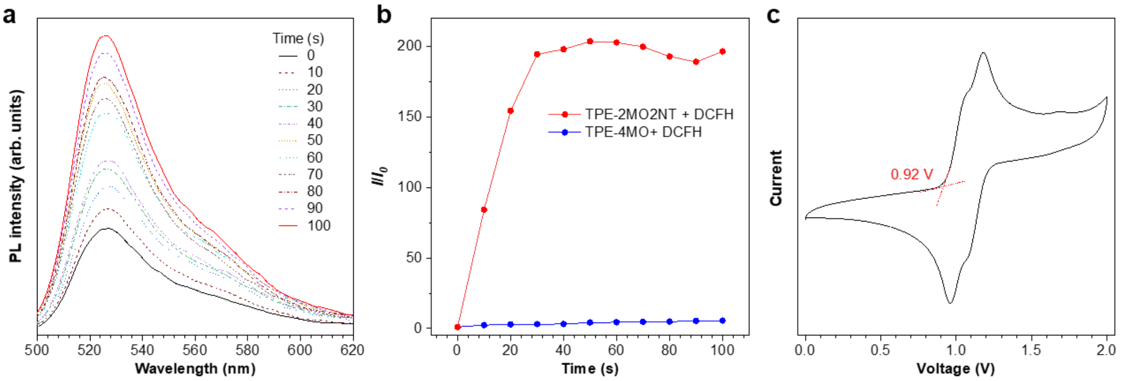


**Supplementary Figure 52. ROS generation ability and CV curve**. (a) PL spectra of DCFH (for overall ROS detection) in the presence of TPE-4MO after exposure to UV light irradiation at different times. (b) Relative changes in PL intensity of DCFH with TPE-4MO or TPE-2MO2NT. (c) Cyclic voltammogram of TPE-4MO in $CH_2Cl_2$ solution.

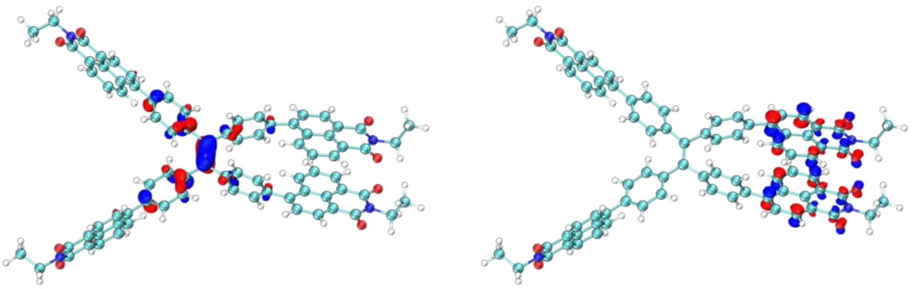

HOMO LUMO

**Supplementary Figure 53. Optimized molecular orbital.** Molecular orbital amplitude plots of HOMO and LUMO of TPE-4NT

**Supplementary Table 6. Summary data of theoretical calculation.** The singlet energy, triplet energy, singlet-triplet energy gap, and SOC coefficient values of TPE-4 NT.

| $T_n$ | Energy (eV) | $S_1^a/T_n$ | $S_1/T_n$ Energy gap | $\xi^b$ ($S_1$, $T_n$ ) ($cm^1$) |
|---|---|---|---|---|
| T1 | 2.049 | $S_1/T_1$ | 1.354 | 0.164 |
| T2 | 2.077 | $S_1/T_2$ | 1.326 | 0.213 |
| T3 | 2.08 | $S_1/T_3$ | 1.323 | 0.066 |
| T4 | 2.102 | $S_1/T_4$ | 1.301 | 0.117 |
| T5 | 2.333 | $S_1/T_5$ | 1.07 | 0.157 |
| T6 | 3.106 | $S_1/T_6$ | 0.297 | 0.085 |
| T7 | 3.159 | $S_1/T_7$ | 0.244 | 0.351 |
| T8 | 3.232 | $S_1/T_8$ | 0.171 | 0.185 |
| T9 | 3.364 | $S_1/T_9$ | 0.039 | 0.075 |
| T10 | 3.398 | $S_1/T_{10}$ | 0.005 | 0.28 |
| T11 | 3.407 | $S_1/T_{11}$ | -0.004 | 0.226 |
| T12 | 3.425 | $S_1/T_{12}$ | -0.022 | 0.14 |

a: $S_1$ = 3.403 eV; b: SOC coefficients ($\xi$).

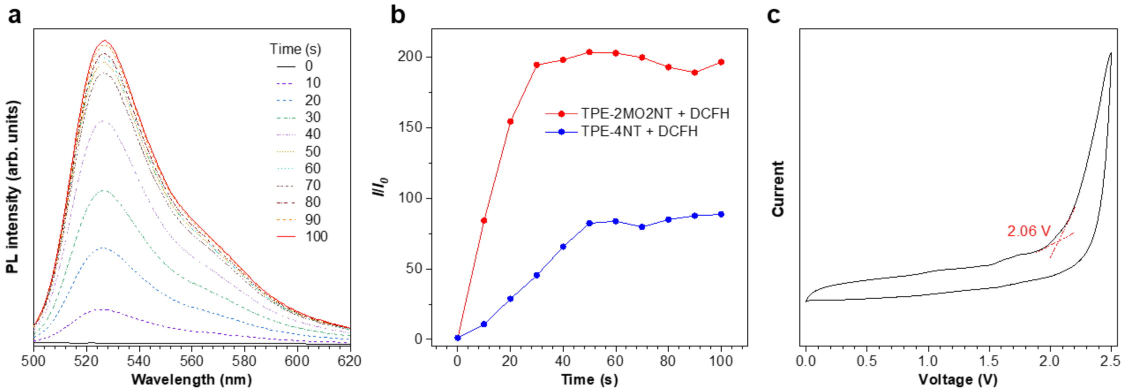


**Supplementary Figure 54. ROS generation ability and CV curve**. (a) PL spectra of DCFH (for overall ROS detection) in the presence of TPE-4NT after exposure to UV light irradiation at different times. (b) Relative changes in PL intensity of DCFH with TPE-4NT or TPE-2MO2NT. (c) Cyclic voltammogram of TPE-4NT in $CH_2Cl_2$ solution.

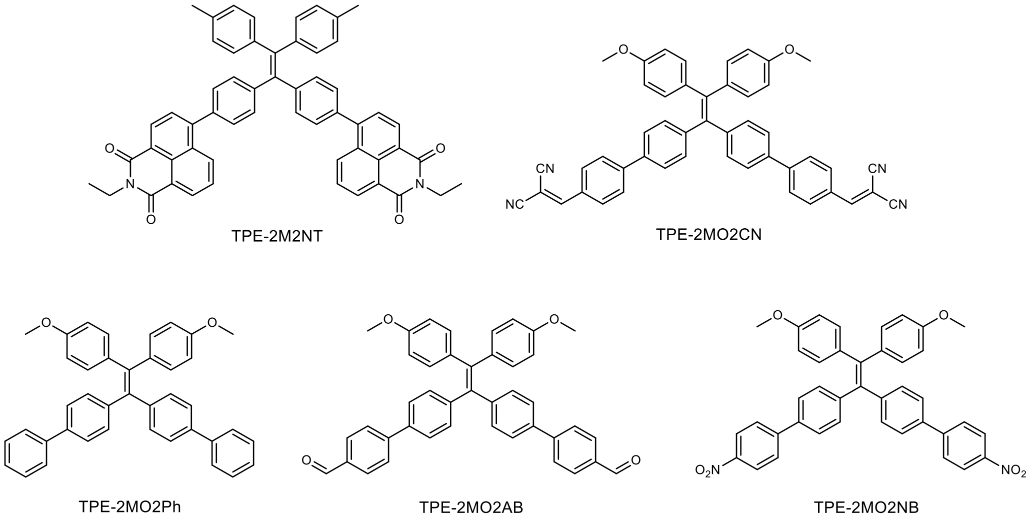


**Supplementary Figure 55. Five contrast molecules.** The chemical structure of five compared compounds.

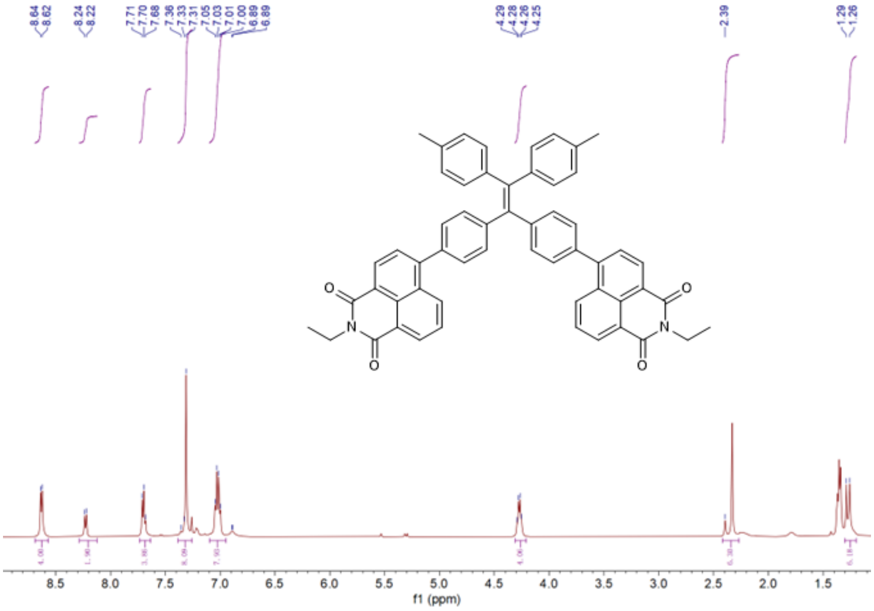


**Supplementary Figure 56. NMR spectrum.** The $^{1}H$ NMR spectrum of TPE-2M2NT in $CDCl_3$.

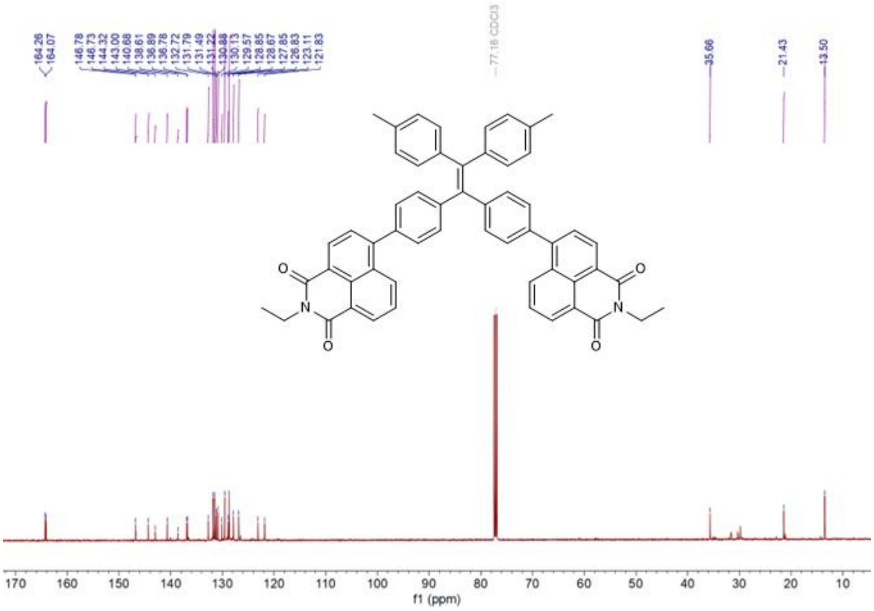


**Supplementary Figure 57. NMR spectrum.** The $^{13}C$ NMR spectrum of TPE-2M2NT in $CDCl_3$.

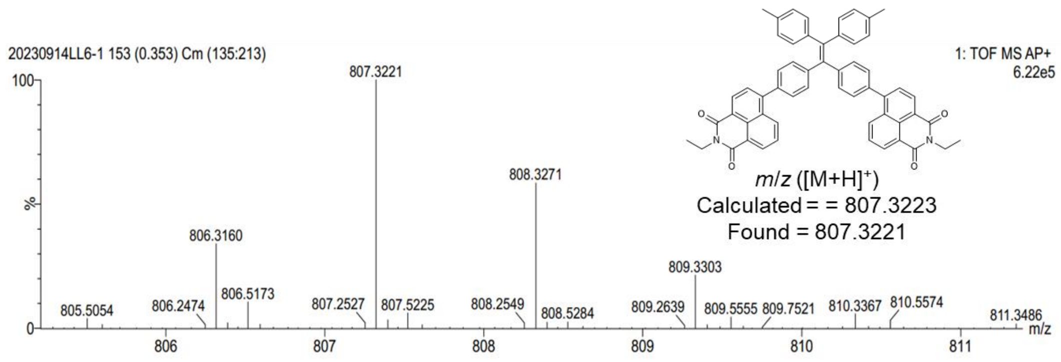


**Supplementary Figure 58. HRMS result.** High-resolution mass spectrum of TPE-2M2NT.

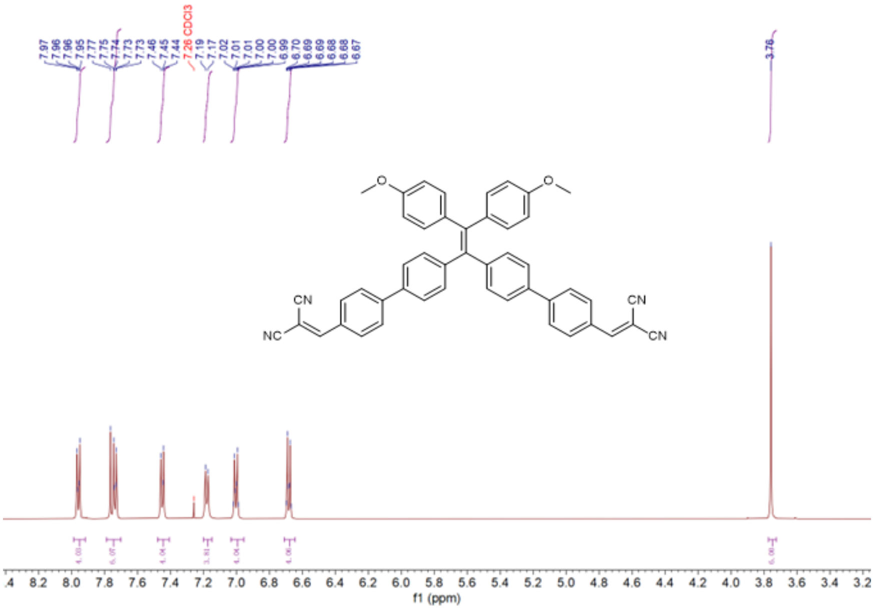


**Supplementary Figure 59. NMR spectrum.** The $^{1}$H NMR spectrum of TPE-2MO2CN in $CDCl_3$.

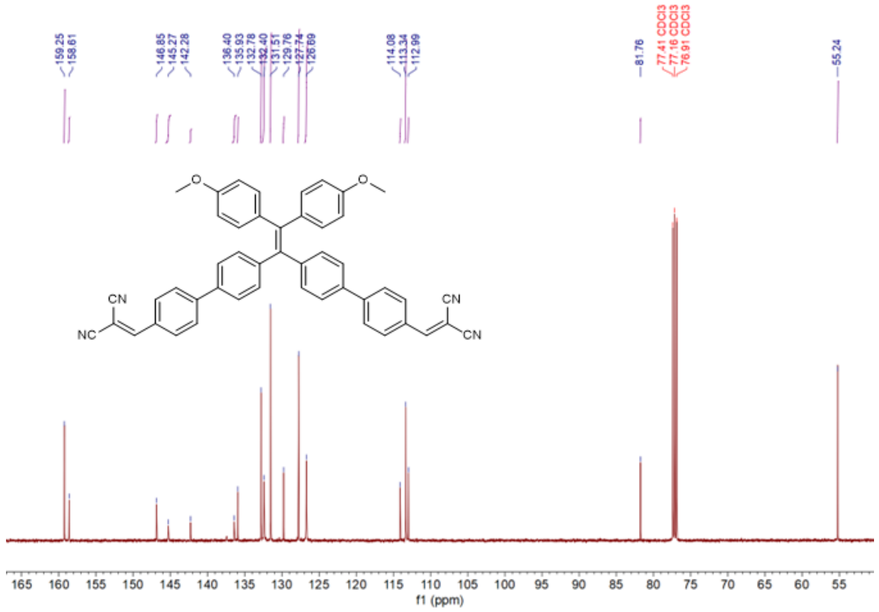


**Supplementary Figure 60. NMR spectrum.** The $^{13}C$ NMR spectrum of TPE-2MO2CN in $CDCl_3$.

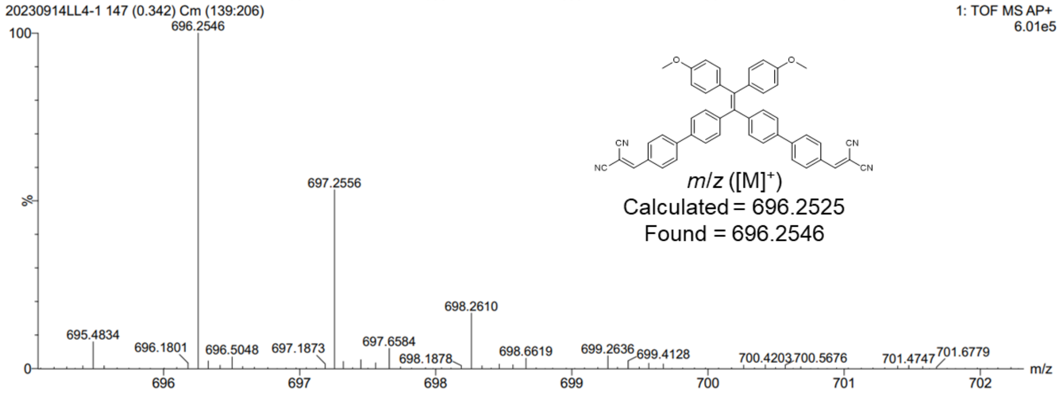


**Supplementary Figure 61. HRMS result.** High-resolution mass spectrum of TPE-2MO2CN.

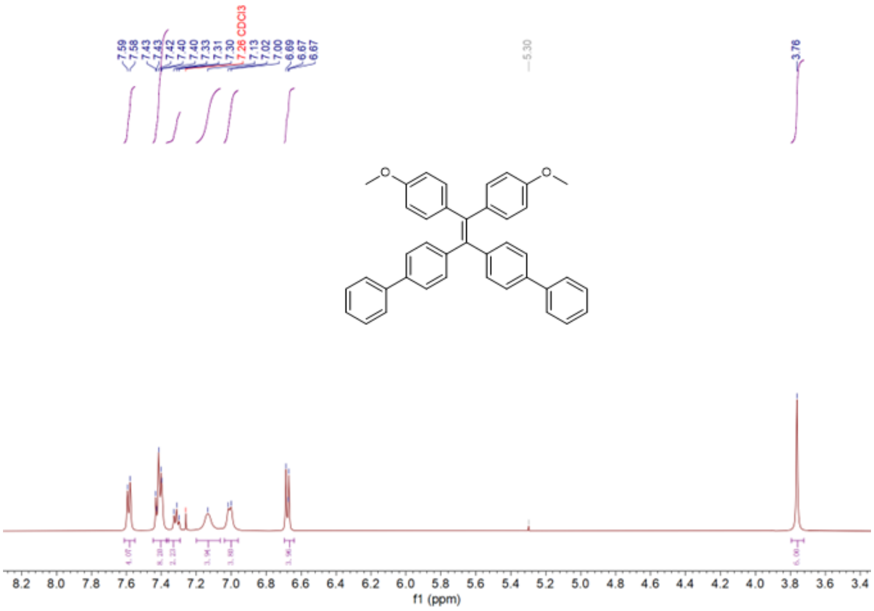


**Supplementary Figure 62. NMR spectrum.** The $^{1}$H NMR spectrum of TPE-2MO2Ph in $CDCl_3$.

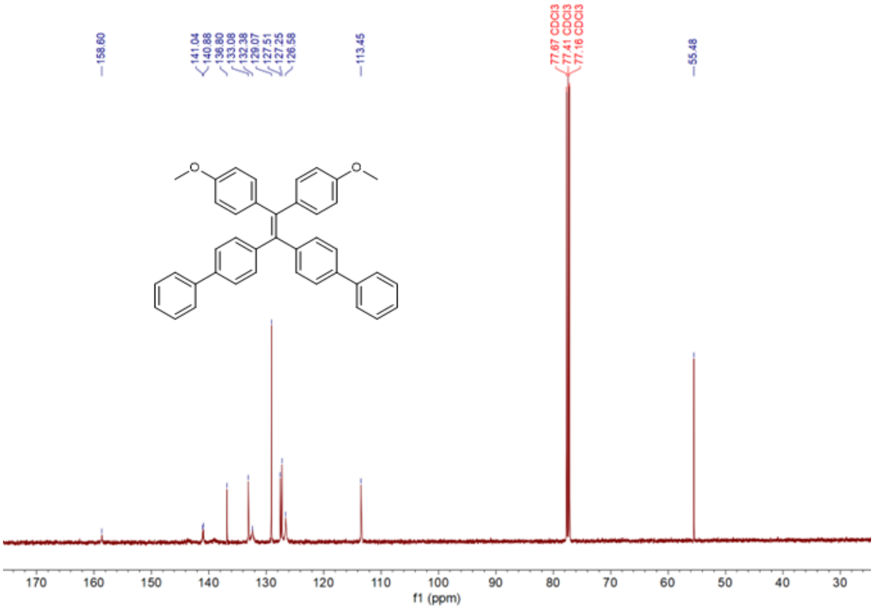


**Supplementary Figure 63. NMR spectrum.** The $^{13}$C NMR spectrum of TPE-2MO2Ph in $CDCl_3$.

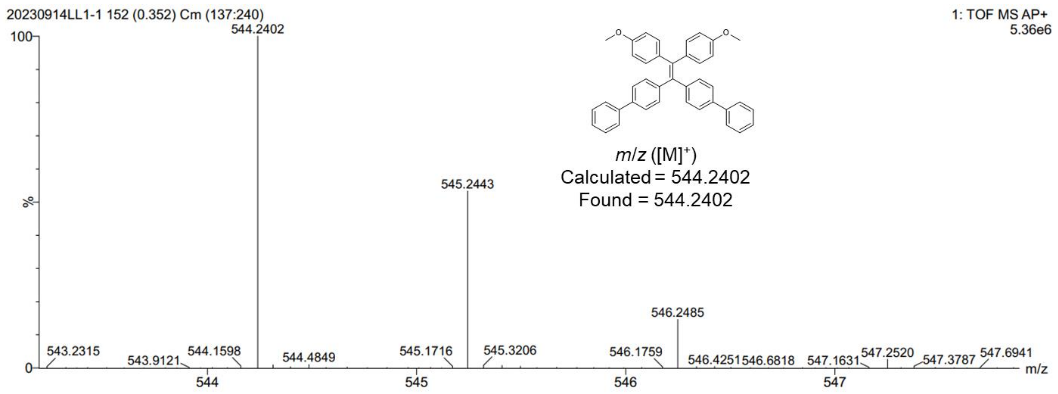


**Supplementary Figure 64. HRMS result.** High-resolution mass spectrum of TPE-2MO2Ph.

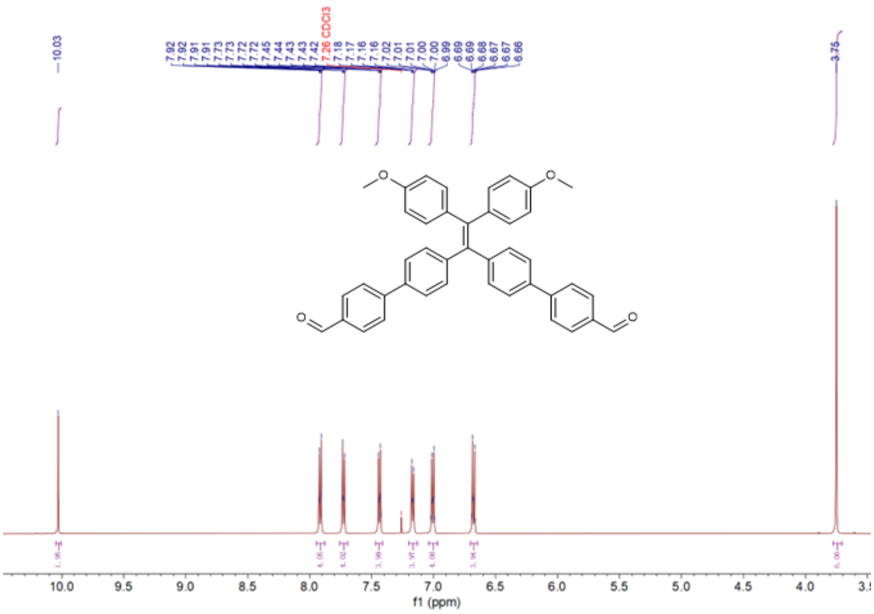


**Supplementary Figure 65. NMR spectrum.** The $^1$H NMR spectrum of TPE-2MO2AB in $CDCl_3$.

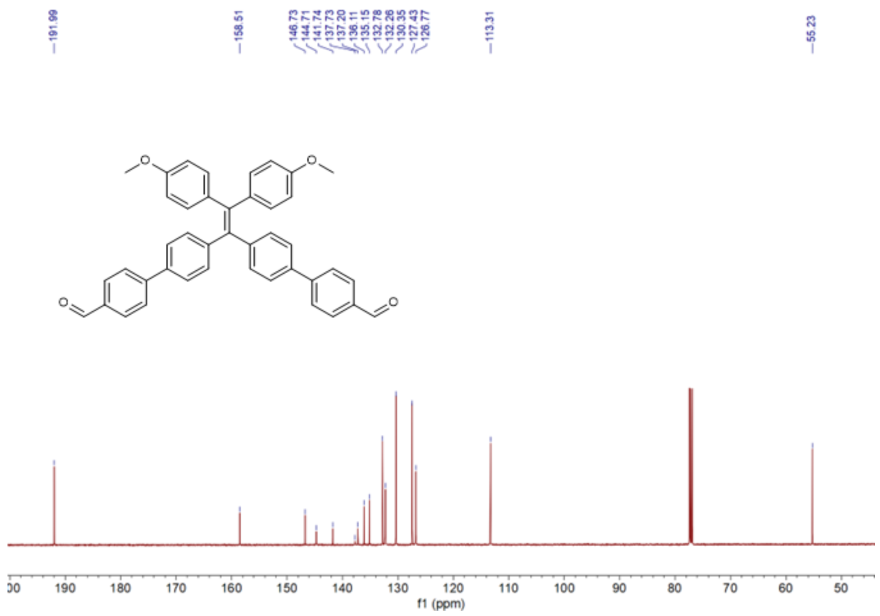


**Supplementary Figure 66. NMR spectrum.** The $^{13}C$ NMR spectrum of TPE-2MO2AB in $CDCl_3$.

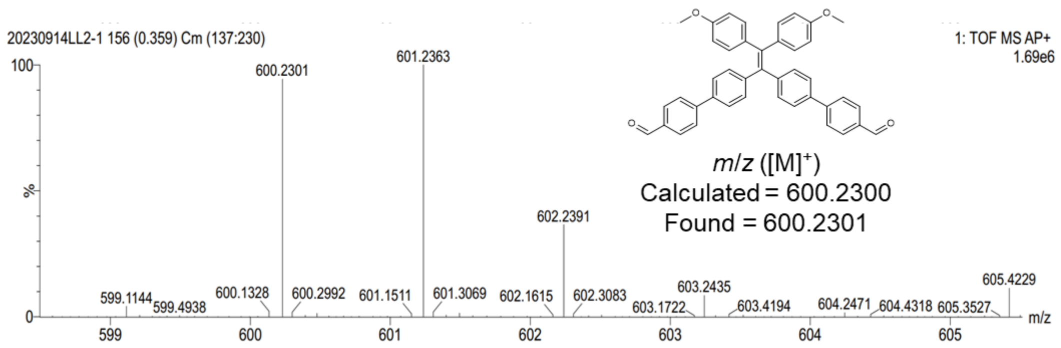


**Supplementary Figure 67. HRMS result.** High-resolution mass spectrum of TPE-2MO2AB.

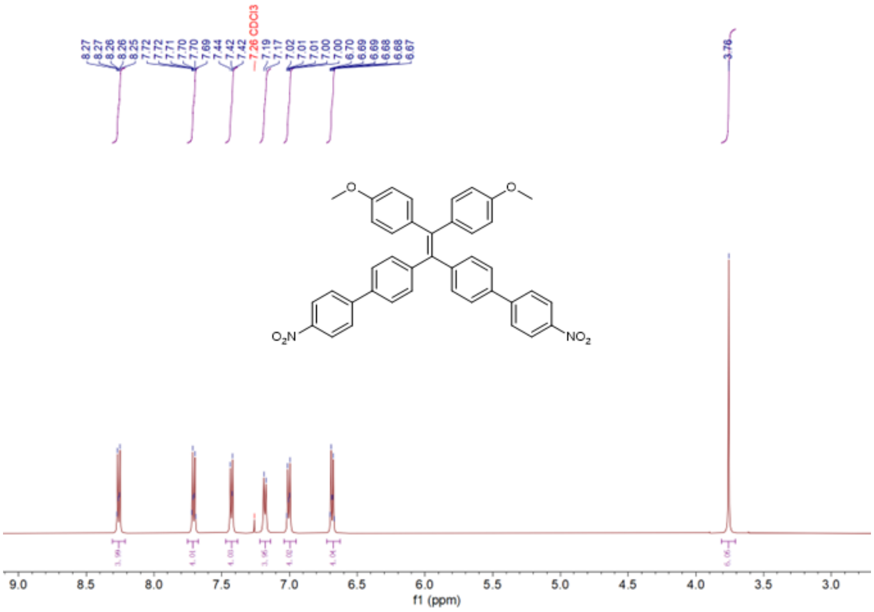


**Supplementary Figure 68. NMR spectrum.** The $^{1}H$ NMR spectrum of TPE-2MO2NB in $CDCl_3$.

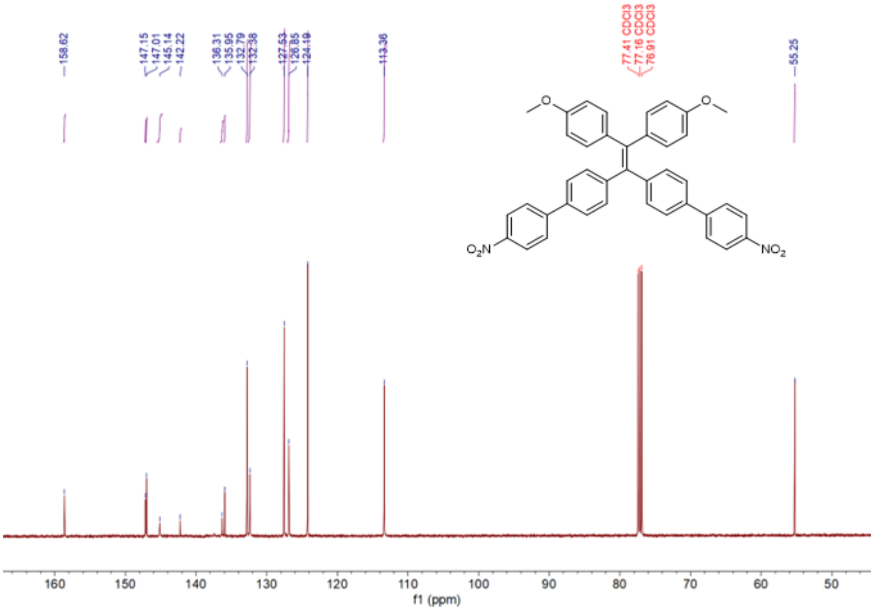


**Supplementary Figure 69. NMR spectrum.** The $^{13}C$ NMR spectrum of TPE-2MO2NB in $CDCl_3$.

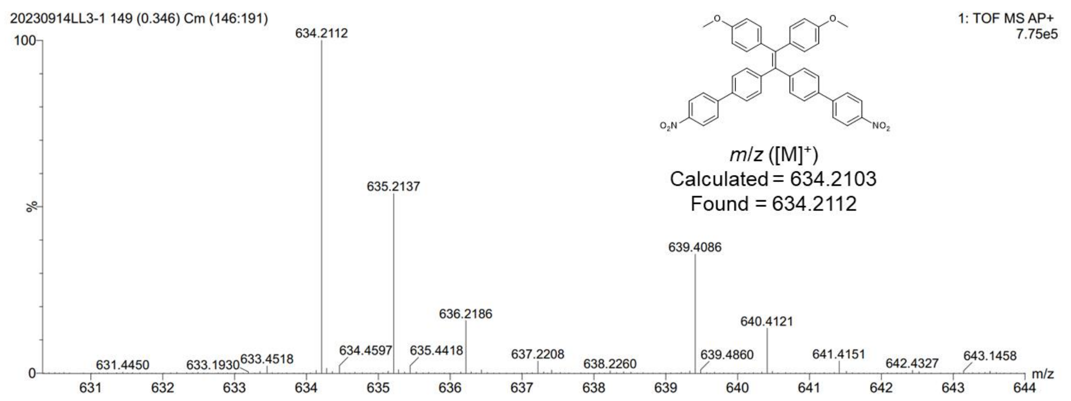


**Supplementary Figure 70. HRMS result.** High-resolution mass spectrum of TPE-2MO2NB.

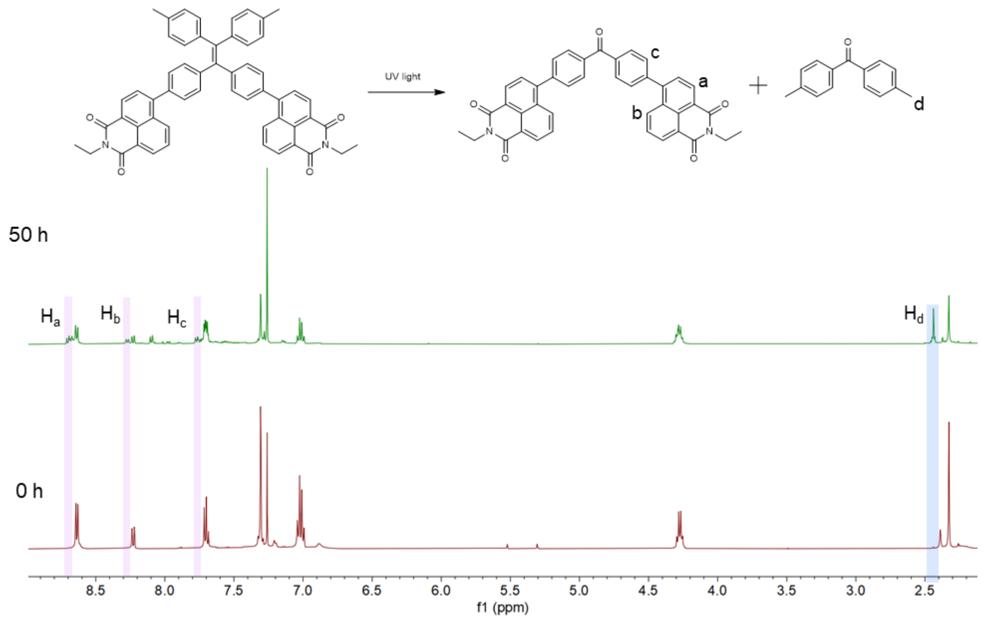


**Supplementary Figure 71. Dynamic NMR spectra of TPE-2M2NT in the illumination process.** $^{1}H$ NMR spectra of TPE-2M2NT in $CDCl_3$ solution before and after under light irradiation. The highlighted new peaks were assigned to the proton of the photoproduct.

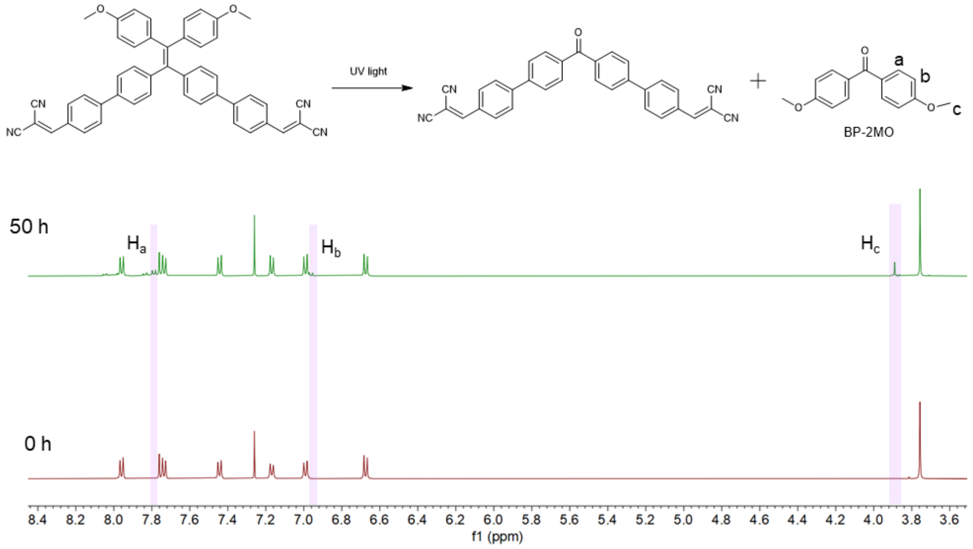


**Supplementary Figure 72. Dynamic NMR spectra of TPE-2MO2CN in the illumination process.** $^{1}$H NMR spectra of TPE-2MO2CN in $CDCl_3$ solution before and after under light irradiation. The highlighted new peaks were assigned to the proton of photoproduct (BP-2MO).

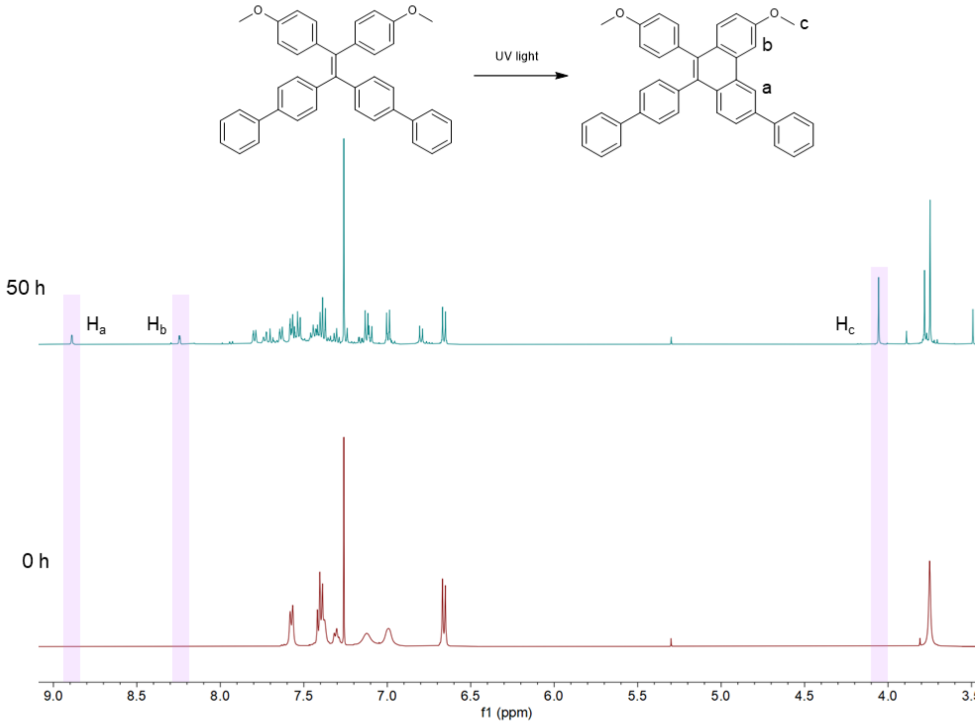


**Supplementary Figure 73. Dynamic NMR spectra of TPE-2MO2Ph in the illumination process.** $^{1}$H NMR spectra of TPE-2MO2Ph in $CDCl_3$ solution before and after under light irradiation. The highlighted new peaks were assigned to the proton of

the photoproduct.

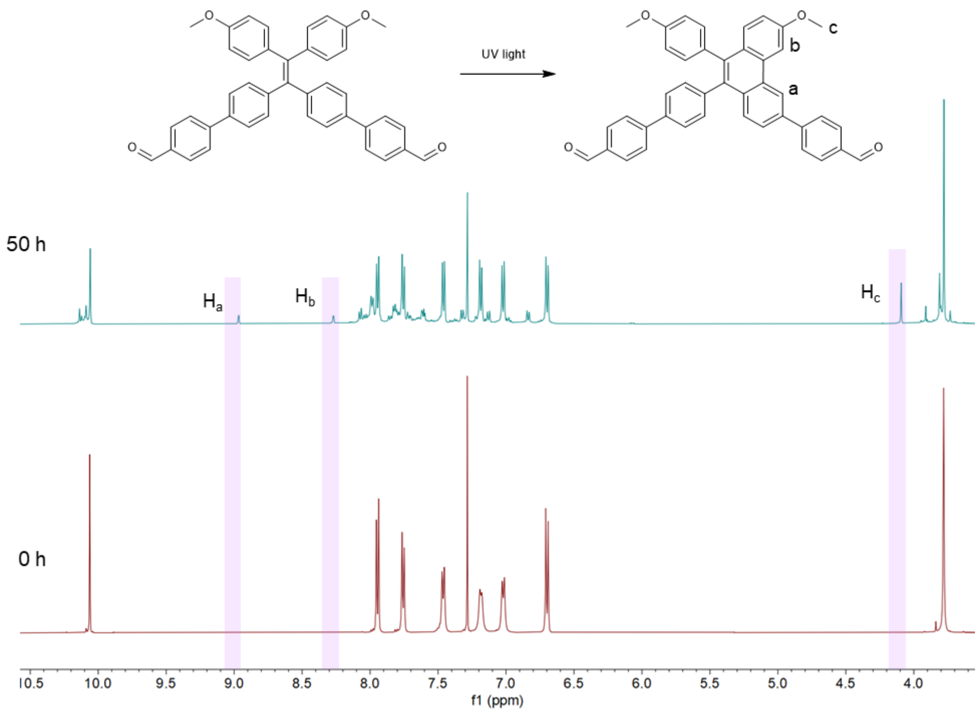


**Supplementary Figure 74. Dynamic NMR spectra of TPE-2MO2AB in the illumination process.** $^1$H NMR spectra of TPE-2MO2AB in $CDCl_3$ solution before and after under light irradiation. The highlighted new peaks were assigned to the proton of the photoproduct.

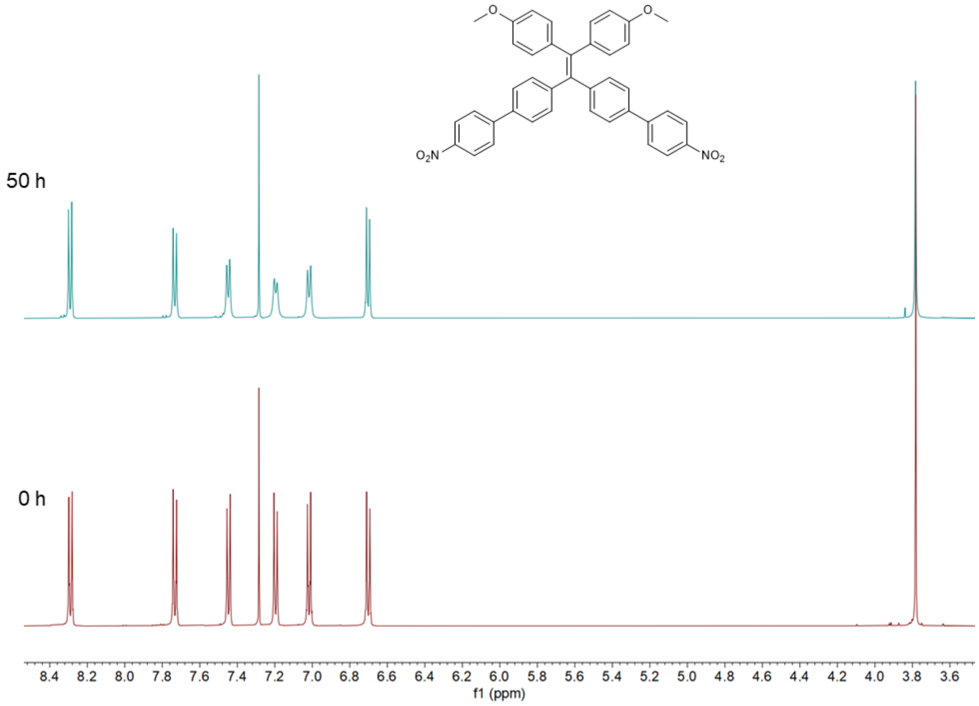

**Supplementary Figure 75. Dynamic NMR spectra of TPE-2MO2NB in the illumination process.** $^{1}$H NMR spectra of TPE-2MO2NB in $CDCl_3$ solution before and after under light irradiation.

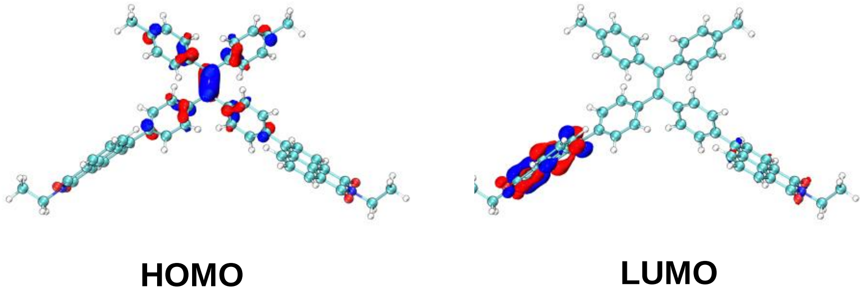


**Supplementary Figure 76. Optimized molecular orbital.** Molecular orbital amplitude plots of HOMO and LUMO of TPE-2M2NT.

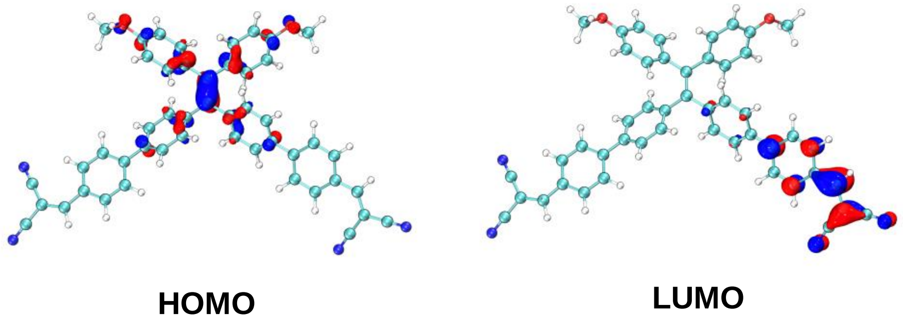


**Supplementary Figure 77. Optimized molecular orbital.** Molecular orbital amplitude plots of HOMO and LUMO of TPE-2MO2CN.

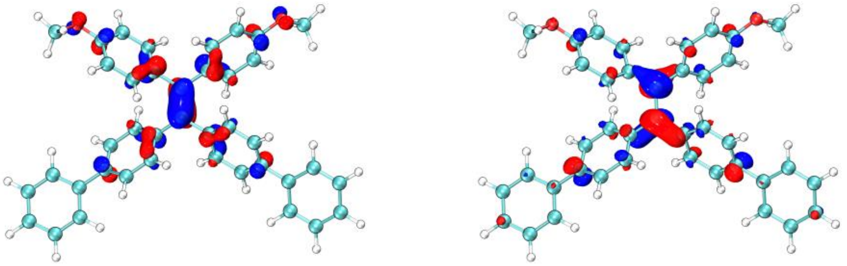

**HOMO** **LUMO**

**Supplementary Figure 78. Optimized molecular orbital.** Molecular orbital amplitude plots of HOMO and LUMO of TPE-2MO2Ph.

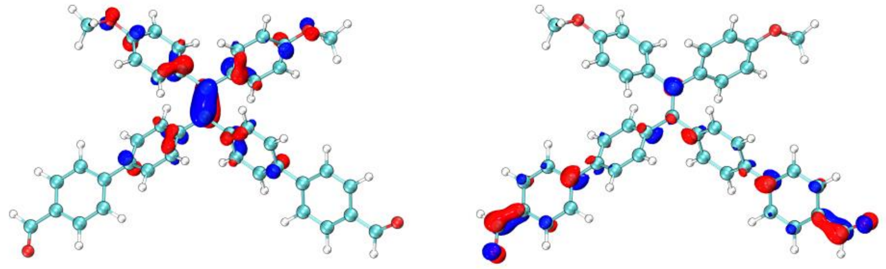

**HOMO** **LUMO**

**Supplementary Figure 79. Optimized molecular orbital.** Molecular orbital amplitude plots of HOMO and LUMO of TPE-2MO2AB.

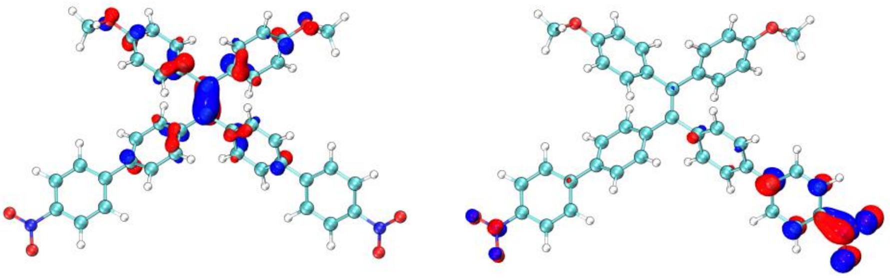

**HOMO** **LUMO**

**Supplementary Figure 80. Optimized molecular orbital.** Molecular orbital amplitude plots of HOMO and LUMO of TPE-2MO2NB.

**Supplementary Table 7. Summary data of the six compounds.**

| Compound | HOMO[a] (eV) | LUMO[a] (eV) | ROS efficiency[b] | λ before UV light[c] | λ after UV light | QY before UV light[d] | QY after UV light |
|---|---|---|---|---|---|---|---|
| TPE-2MO2NT | -6.62 | -1.67 | 203.63 | 582 | 452 | 42.5 | 13.6 |
| TPE-2M2NT | -6.81 | -1.68 | 180.87 | 536 | 485 | 40.3 | 12.2 |
| TPE-2MO2CN | -6.60 | -2.02 | 45.90 | 668 | 650 | 12.3 | 0.28 |
| TPE-2MO2Ph | -6.48 | -0.64 | 3.85 | 519 | 483 | 48.01 | 0.06 |
| TPE-2MO2AB | -6.50 | -1.20 | 138.30 | 539 | 481 | 34.03 | 0.08 |
| TPE-2MO2NB | -6.61 | -1.71 | 288.46 | 621 | 618 | 7.3 | 1.16 |

[a]: HOMO and LUMO levels were calculated by DFT; [b]: Relative PL intensity of DCFH with compound before and after irradiation, respectively; [c]: Emission peak of compound in THF/$H_2O$ ($f_w$ = 90%) aggregate mixtures; [d]: Quantum yield of compound in THF/$H_2O$ ($f_w$ = 90%) aggregate mixtures.

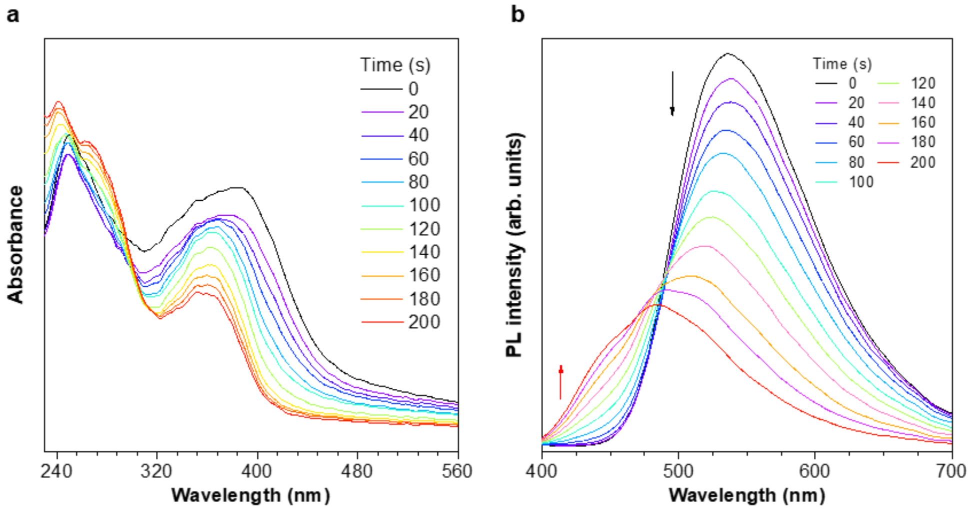


**Supplementary Figure 81. Photophysical spectra changes of TPE-2M2NT under light irradiation.** Absorption and PL spectra of TPE-2M2NT in THF/$H_2O$ ($f_w$ = 90%) aggregate mixtures over time upon 365 nm UV irradiation. Molecular concentration: $10^{-5}$ M. $\lambda_{ex}$ = 380 nm.

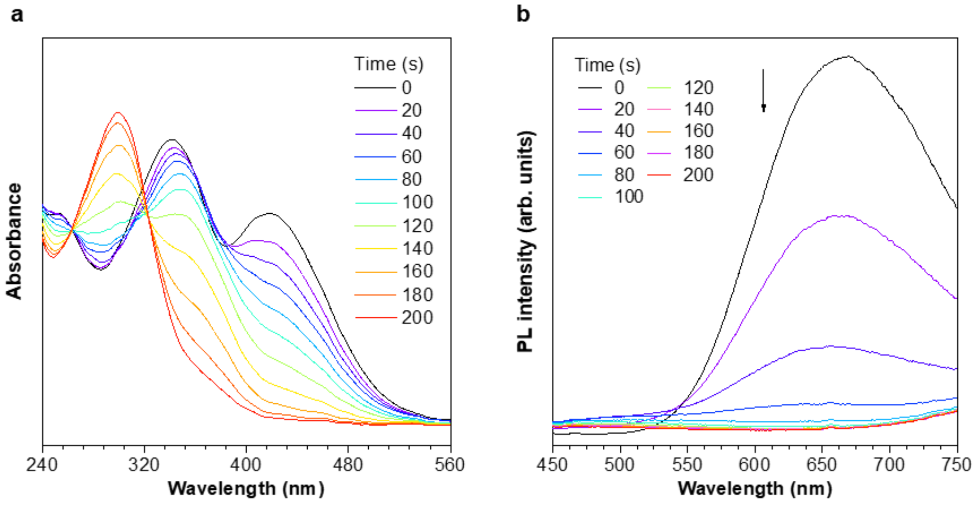


**Supplementary Figure 82. Photophysical spectra changes of TPE-2MO2CN under light irradiation.** Absorption and PL spectra of TPE-2MO2CN in THF/$H_2O$ ($f_w$ = 90%) aggregate mixtures over time upon 365 nm UV irradiation. Molecular concentration: $10^{-5}$ M. $\lambda_{ex}$ = 420 nm.

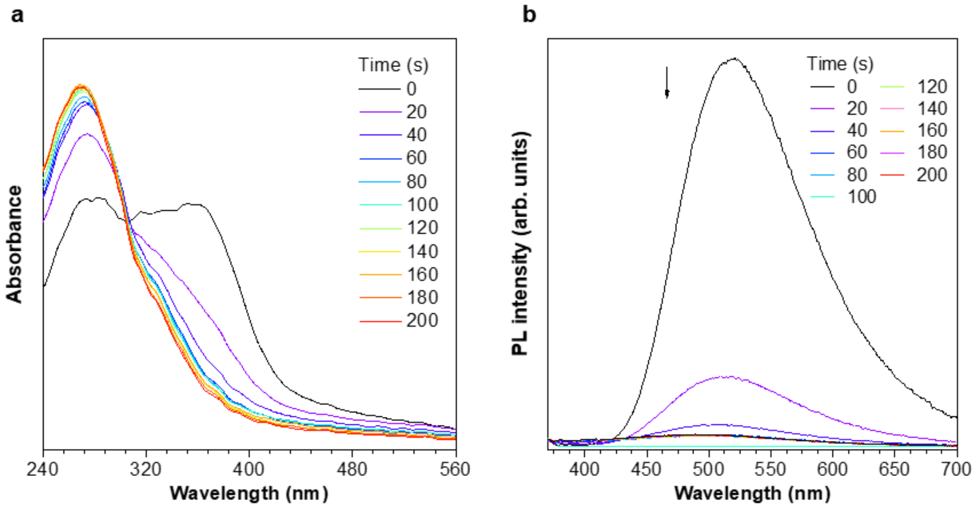


**Supplementary Figure 83. Photophysical spectra changes of TPE-2MO2Ph under light irradiation.** Absorption and PL spectra of TPE-2MO2Ph in THF/$H_2O$ ($f_w$ = 90%) aggregate mixtures over time upon 365 nm UV irradiation. Molecular concentration: $10^{-5}$ M. $\lambda_{ex}$ = 365 nm.

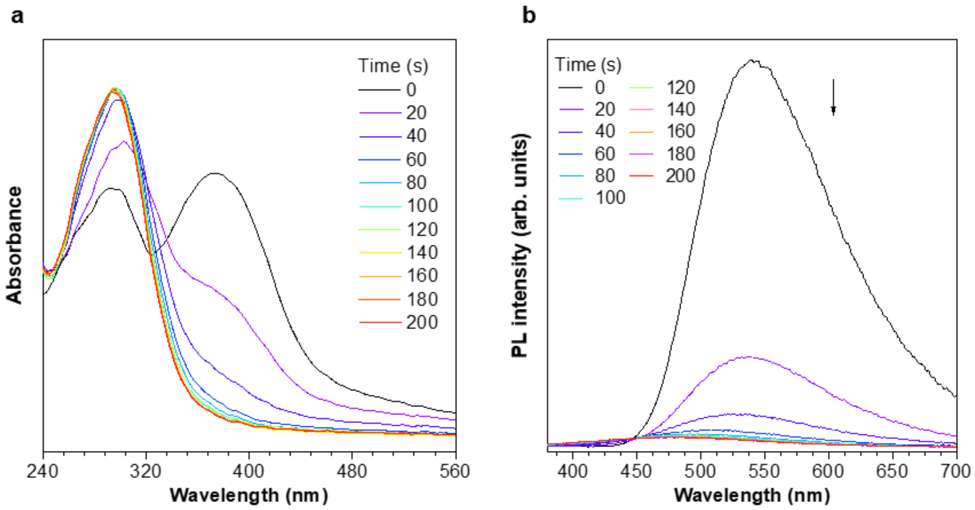


**Supplementary Figure 84. Photophysical spectra changes of TPE-2MO2AB under light irradiation.** Absorption and PL spectra of TPE-2MO2AB in THF/$H_2O$ ($f_w$ = 90%) aggregate mixtures over time upon 365 nm UV irradiation. Molecular concentration: $10^{-5}$ M. $\lambda_{ex}$ = 380 nm.

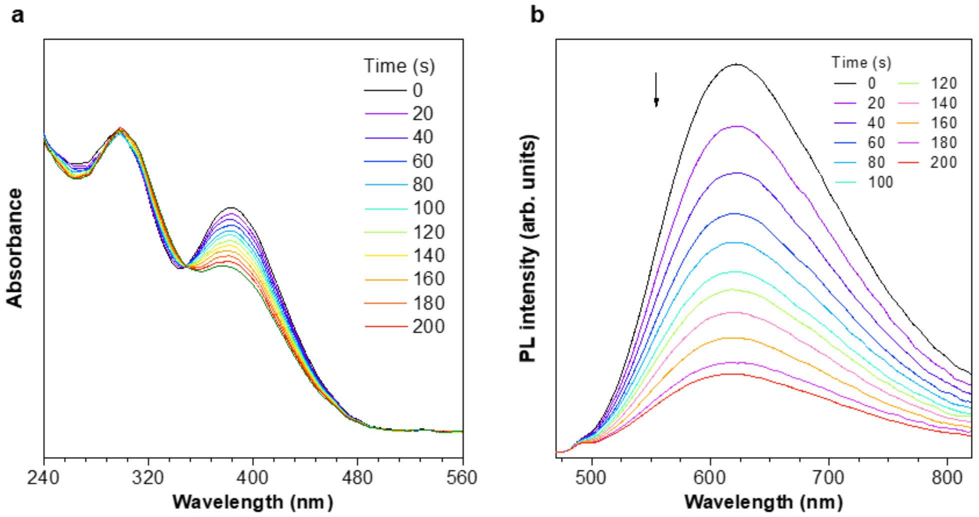


**Supplementary Figure 85. Photophysical spectra changes of TPE-2MO2NB under light irradiation.** Absorption and PL spectra of TPE-2MO2NB in THF/$H_2O$ ($f_w$ = 90%) aggregate mixtures over time upon 365 nm UV irradiation. Molecular concentration: $10^{-5}$ M. $\lambda_{ex}$ = 420 nm.

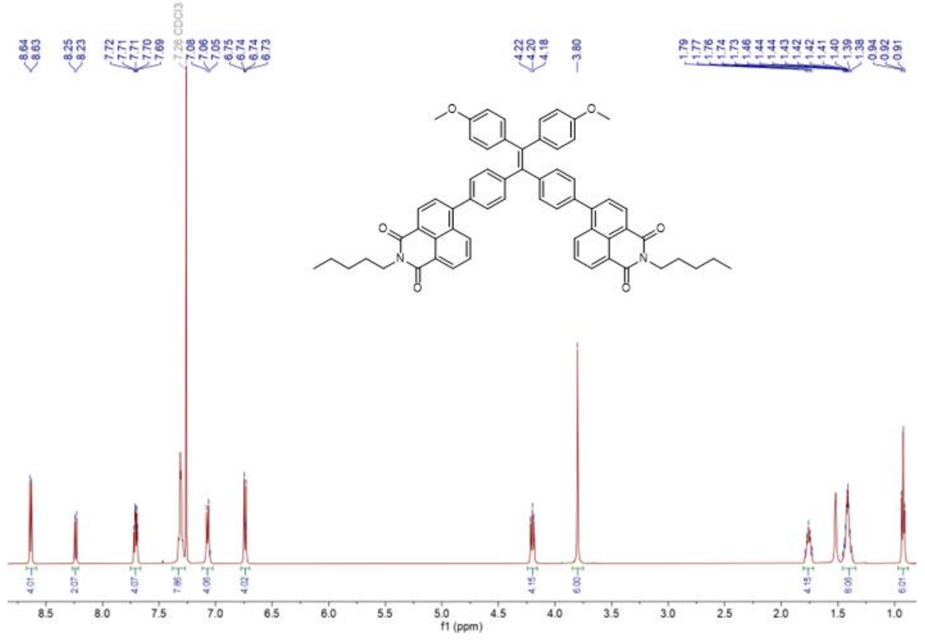


**Supplementary Figure 86. NMR spectrum.** $^{1}$H NMR spectrum of TPE-MN-C5 in $CDCl_3$.

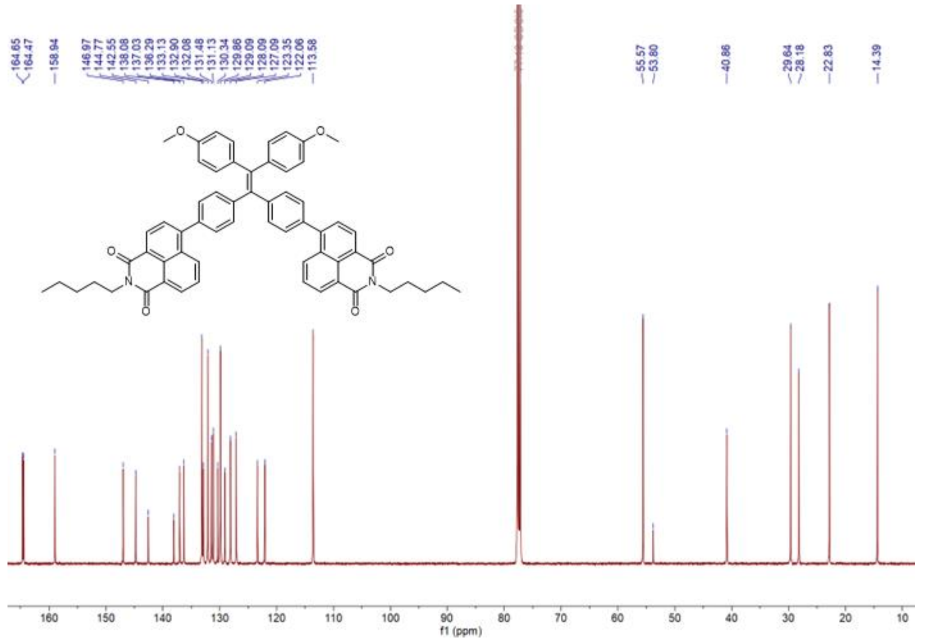


**Supplementary Figure 87. NMR spectrum.** $^{13}$C NMR spectrum of TPE-MN-C5 in $CDCl_3$.

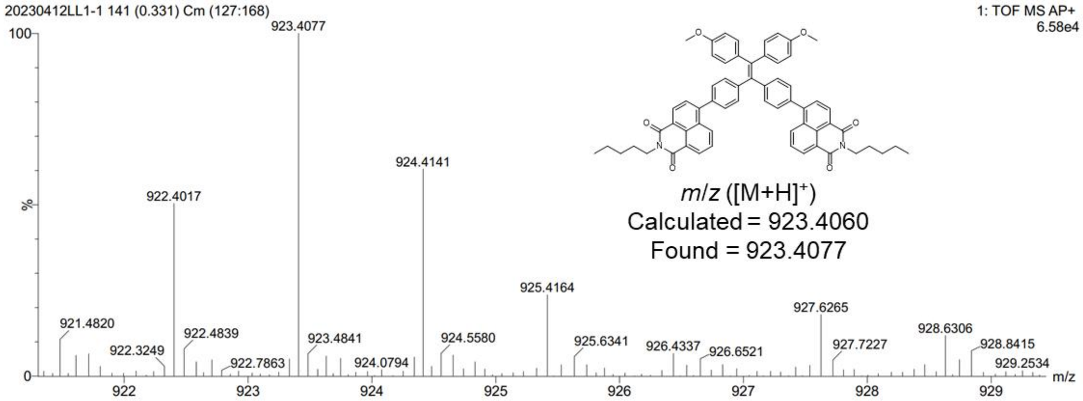


**Supplementary Figure 88. HRMS result.** High-resolution mass spectrum of TPE-MN-C5.

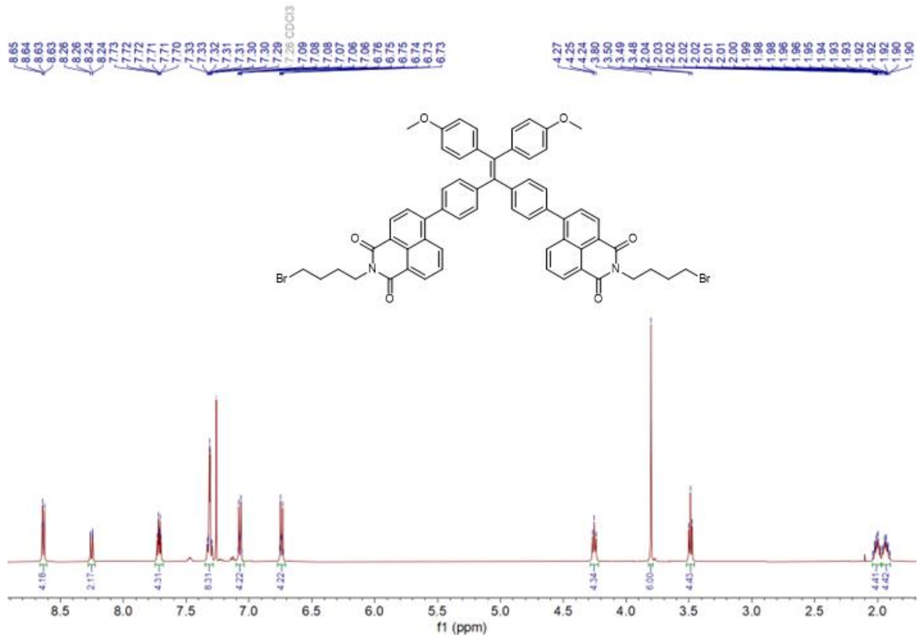


**Supplementary Figure 89. NMR spectrum.** $^{1}$H NMR spectrum of TPE-MN-Br in $CDCl_3$.

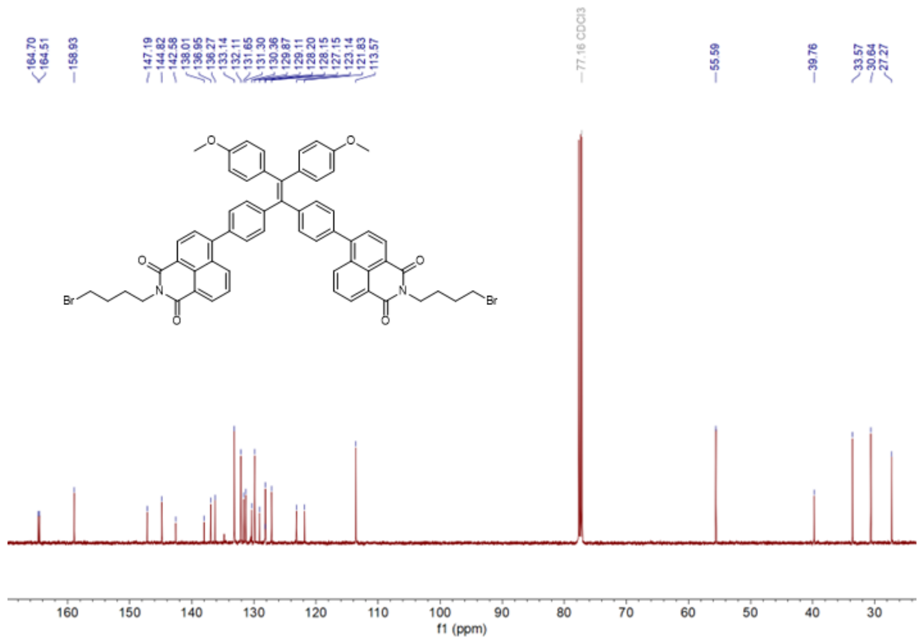

**Supplementary Figure 90. NMR spectrum.** $^{13}$C NMR spectrum of TPE-MN-Br in $CDCl_3$.

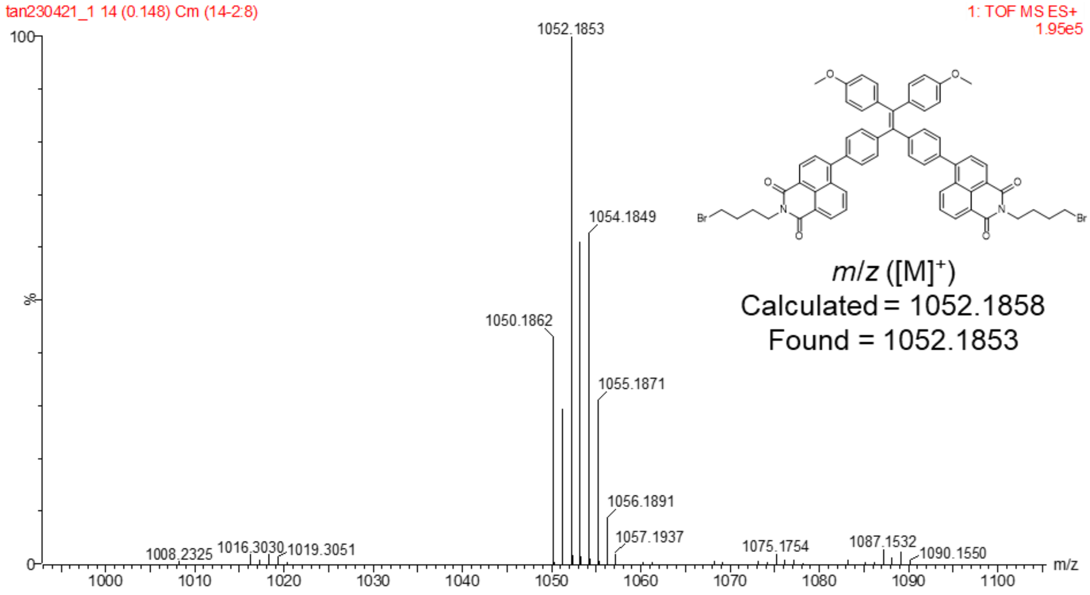


**Supplementary Figure 91. HRMS result.** High-resolution mass spectrum of TPE-MN-Br.

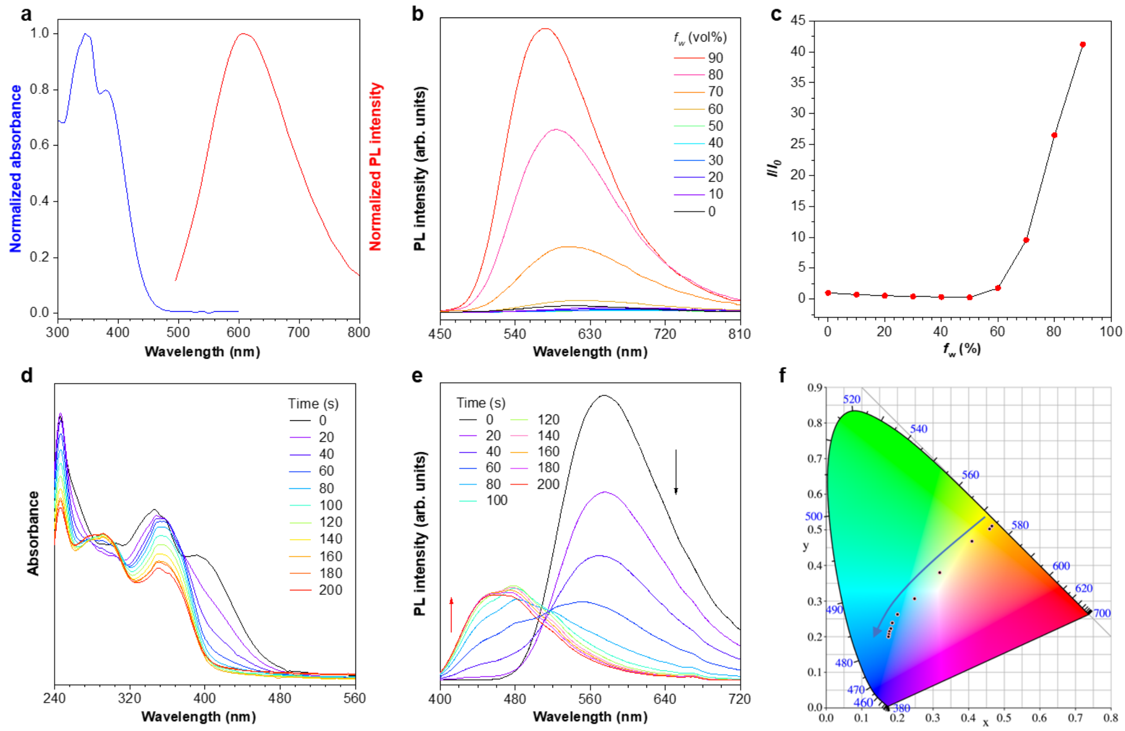


**Supplementary Figure 92. Photophysical property of TPE-MN-C5.** (a) Absorption and PL spectra of TPE-MN-C5 in THF solution. (b) PL spectra of TPE-MN-C5 in THF/$H_2O$ mixtures with different water fractions ($f_w$). (c) Plots of relative PL intensity ($I/I_0$) versus water fractions. (d) Absorption spectra, (e) PL spectra, and (f) CIE 1931 chromaticity diagram of TPE-MN-C5 in THF/$H_2O$ ($f_w$ = 90%) aggregate mixtures with different times upon 365 nm UV irradiation. Molecular concentration: $10^{-5}$ M; $\lambda_{ex}$ = 380 nm.

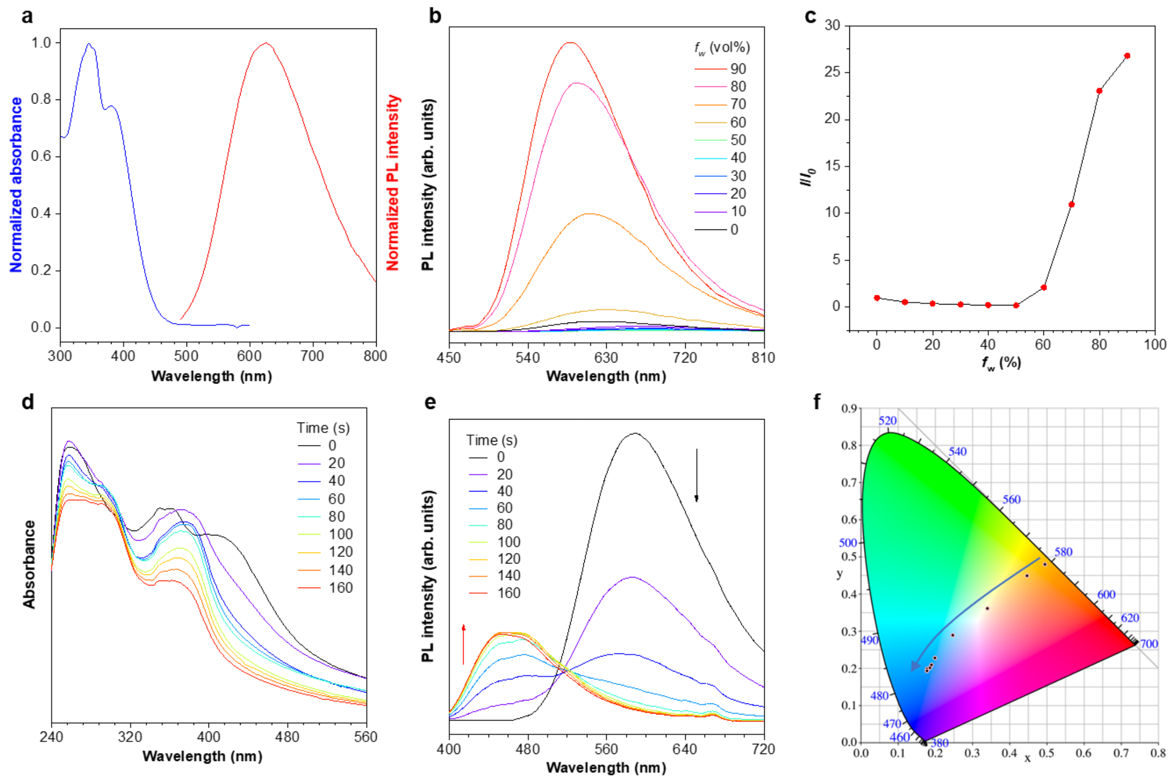


**Supplementary Figure 93. Photophysical property of TPE-MN-Br.** (a) Absorption and PL spectra of TPE-MN-Br in THF solution. (b) PL spectra of TPE-MN-Br in THF/$H_2O$ aggregate mixtures with different water fractions ($f_w$). (c) Plots of relative PL intensity ($I/I_0$) versus water fractions. (d) Absorption spectra, (e) PL spectra, and (f) CIE 1931 chromaticity diagram of TPE-MN-Br in THF/$H_2O$ ($f_w$ = 90%) aggregate mixtures with different times upon 365 nm UV irradiation. Molecular concentration: $10^{-5}$ M; $\lambda_{ex}$ = 380 nm.

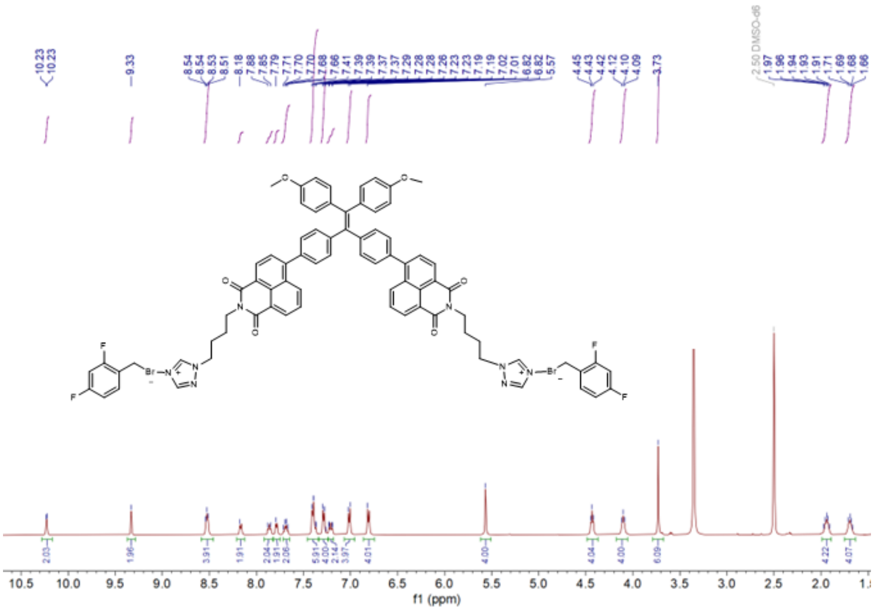


**Supplementary Figure 94. NMR spectrum.** The $^{1}H$ NMR spectrum of TPE-MN-TA in DMSO-d6.

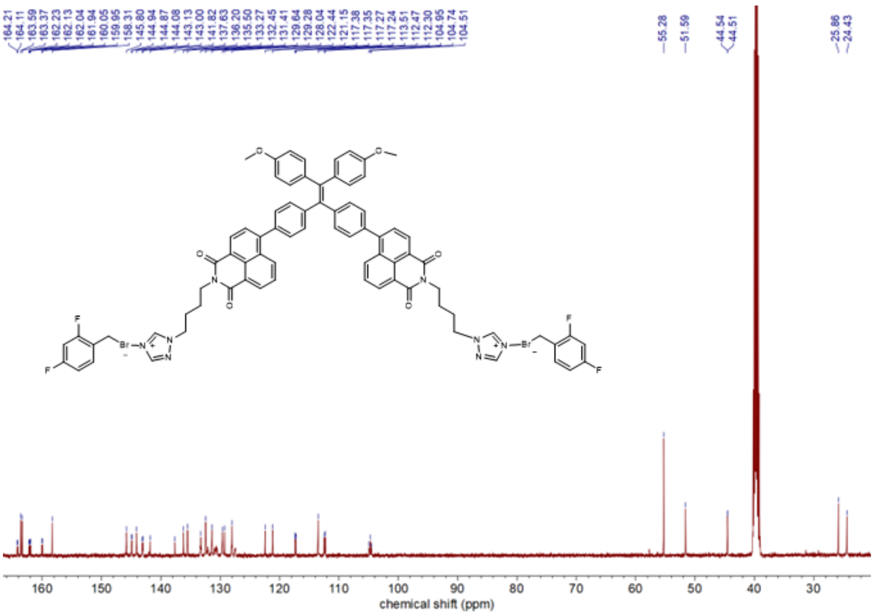


**Supplementary Figure 95. NMR spectrum.** The $^{13}C$ NMR spectrum of TPE-MN-TA in DMSO-d6.

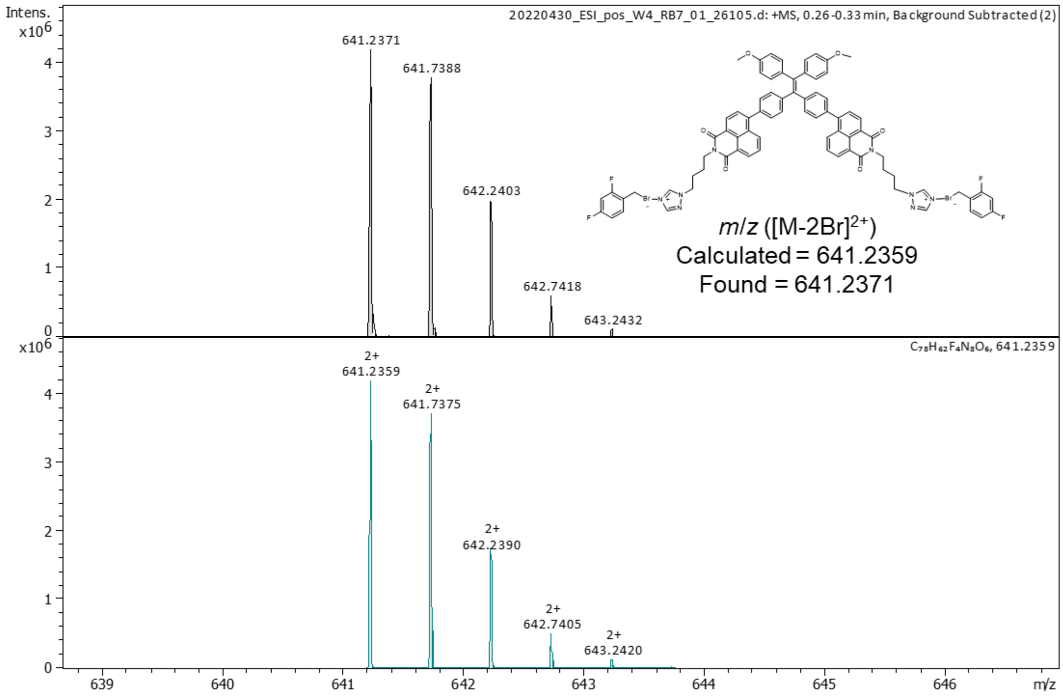


**Supplementary Figure 96. HRMS result.** HRMS result. High-resolution mass spectrum of TPE-MN-TA.

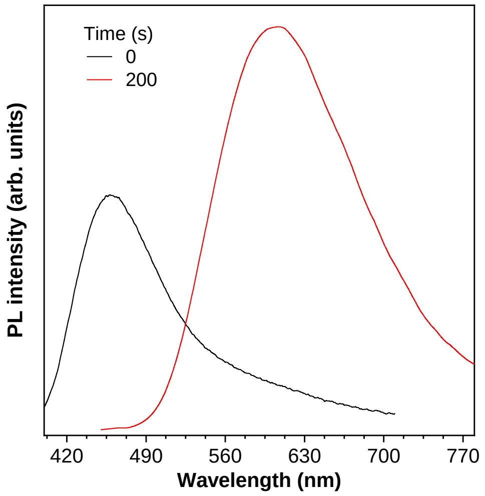


**Supplementary Figure 97. PL spectra changes of TPE-MN-TA under light irradiation.** PL spectra of TPE-MN-TA (10 μM) in DMSO/$H_2O$ ($f_w$ = 90%) aggregate mixtures before and after 200 s light irradiation. Before: $\lambda_{ex}$ = 350 nm; After: $\lambda_{ex}$ = 450 nm.

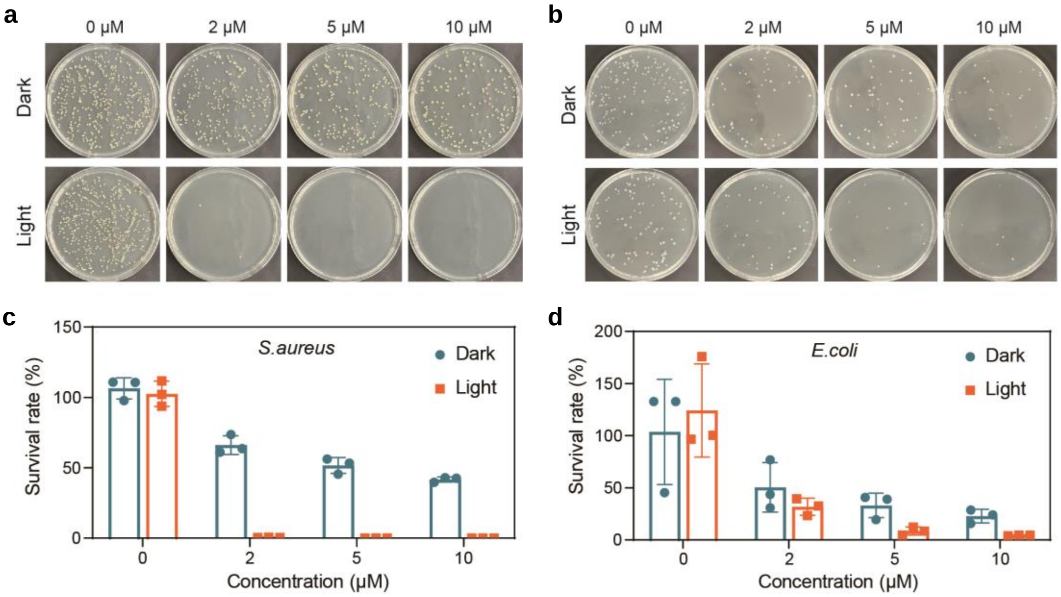


**Supplementary Figure 98. Antibacterial results.** Antibacterial activity of TPE-MN-TA toward (a) G (+) bacteria *S. aureus* and (b) G(-) bacteria *E.coli*. The statistical survival rate of (c) *S. aureus* and (d) *E.coli* exposed to AIEgens (0–10 μM) with/without white light (20 mW $cm^{-2}$ ). Data are presented as mean ± SD (n = 3 independent experiments).

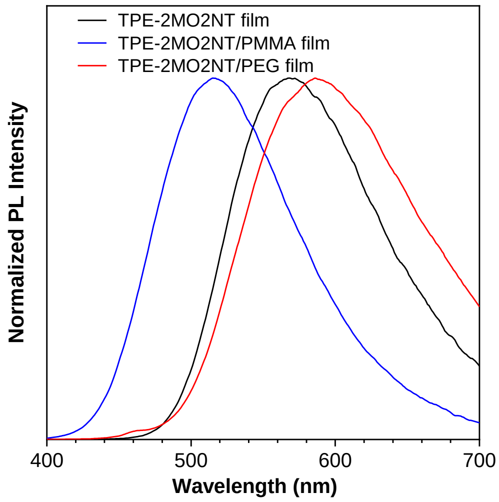


**Supplementary Figure 99. PL spectra of TPE-2MO2NT in film state**. Normalized PL spectra of the TPE-2MO2NT, TPE-2MO2NT/PMMA, and TPE-2MO2NT/PEG film. $\lambda_{ex}$ = 380 nm for TPE-2MO2NT film; $\lambda_{ex}$ = 350 nm for TPE-2MO2NT/PMMA film; $\lambda_{ex}$ = 420 nm for TPE-2MO2NT/PEG film.

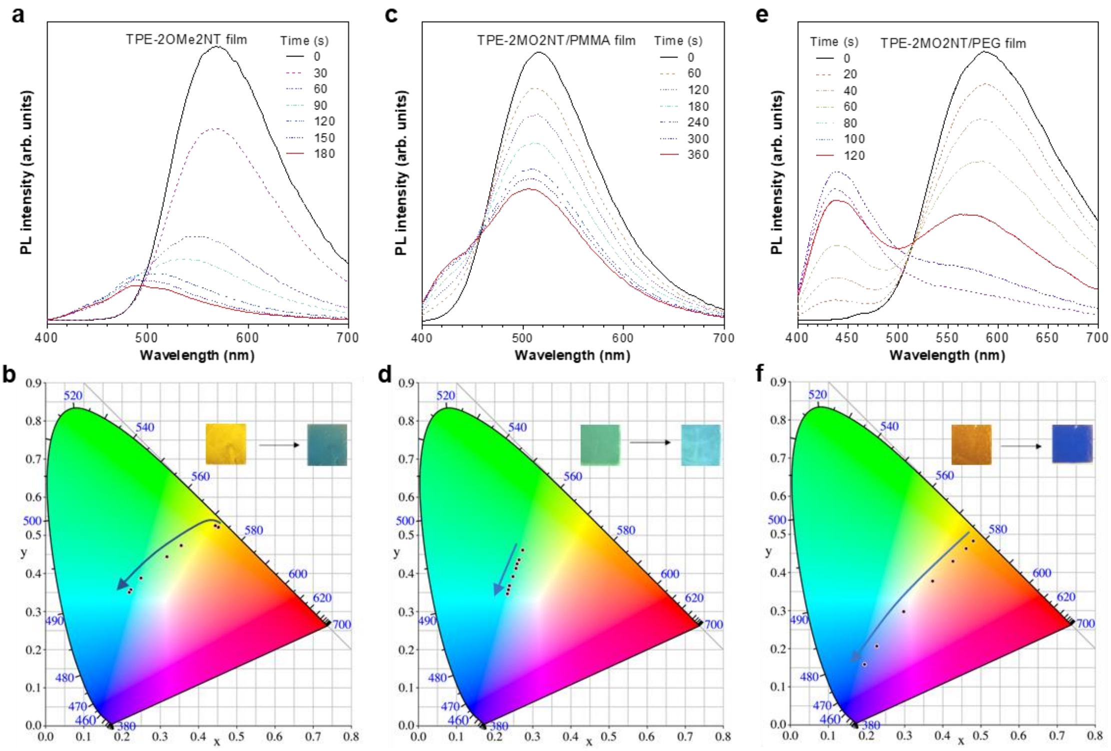


**Supplementary Figure 100. PL spectra change of photoreaction in three film states.** (a) PL spectra and (b) CIE 1931 chromaticity diagram of TPE-2MO2NT film with different times upon 365 nm UV irradiation. (c) PL spectra and (d) CIE 1931 chromaticity diagram of TPE-2MO2NT/PMMA film with different times upon 365 nm UV irradiation. (e) PL spectra and (f) CIE 1931 chromaticity diagram of TPE-2MO2NT /PEG film with different times upon 365 nm UV irradiation. $\lambda_{ex}$ = 380 nm for TPE-2MO2NT film; $\lambda_{ex}$ = 350 nm for TPE-2MO2NT/PMMA film; $\lambda_{ex}$ = 420 nm for TPE-2MO2NT/PEG film.

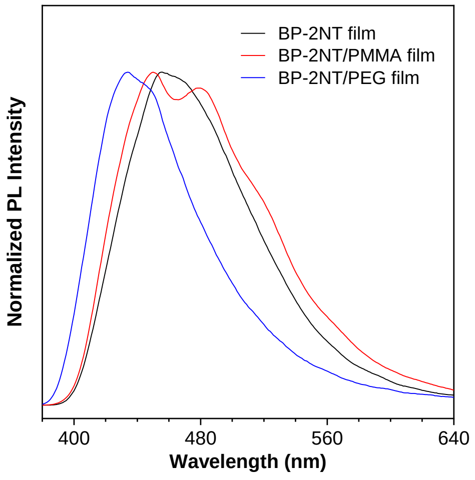

**Supplementary Figure 101. PL spectra of BP-2NT in film state**. Normalized PL spectra of the BP-2NT, BP-2NT/PMMA, and BP-2NT/PEG film. $\lambda_{ex}$ = 300 nm.

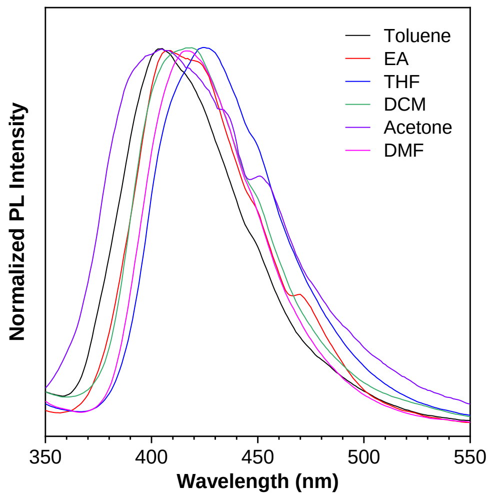


**Supplementary Figure 102. PL spectra of BP-2NT in solution state**. Normalized PL spectra of BP-2NT in different polar solutions.

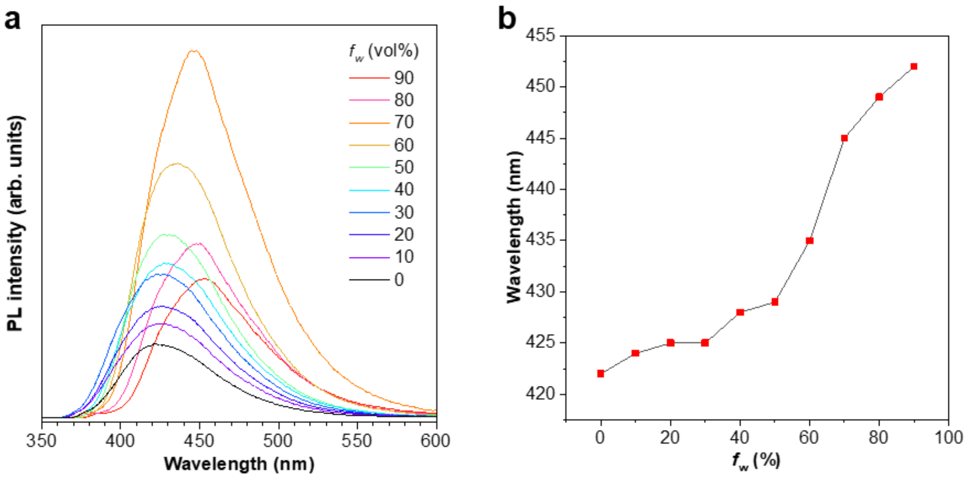


**Supplementary Figure 103. PL intensity and wavelength changes of BP-2NT.** (a) PL spectra of BP-2NT in THF/$H_2O$ solution-aggregates mixtures with different water fractions. (b) Plots of wavelength versus water fractions. Molecular concentration: $10^{-5}$ M; $\lambda_{ex}$ = 340 nm; $I_0$ = emission intensity in THF ($f_w$ = 0%).
