# Supplementary material for: Multiple photofluorochromic luminogens via catalyst-free alkene oxidative cleavage photoreaction for dynamic 4D codes encryption
Source: Nat Commun. 2024 May 31;15:4647. doi: 10.1038/s41467-024-49033-2 (PMC11143217; doi:10.1038/s41467-024-49033-2)
Supplement: Supplementary file 3 — Description of Additional Supplementary Files [file 41467_2024_49033_MOESM3_ESM.pdf]

Description of Additional Supplementary Files

File Name: Supplementary Data 1
Description: Cartesian coordinates of optimized molecular geometry.
